# Deep Insights Into the Plastome Evolution and Phylogenetic Relationships of the Tribe Urticeae (Family Urticaceae)

**DOI:** 10.3389/fpls.2022.870949

**Published:** 2022-05-20

**Authors:** Catherine A. Ogoma, Jie Liu, Gregory W. Stull, Moses C. Wambulwa, Oyetola Oyebanji, Richard I. Milne, Alexandre K. Monro, Ying Zhao, De-Zhu Li, Zeng-Yuan Wu

**Affiliations:** ^1^Germplasm Bank of Wild Species, Kunming Institute of Botany, Chinese Academy of Sciences, Kunming, China; ^2^University of Chinese Academy of Sciences, Beijing, China; ^3^Key Laboratory of Biodiversity and Biogeography, Kunming Institute of Botany, Chinese Academy of Sciences, Kunming, China; ^4^Department of Life Sciences, School of Science and Computing, South Eastern Kenya University, Kitui, Kenya; ^5^School of Biological Sciences, Institute of Molecular Plant Sciences, University of Edinburgh, Edinburgh, United Kingdom; ^6^Royal Botanic Gardens, Kew, Richmond, United Kingdom

**Keywords:** Urticaceae *s.l.*, chloroplast structural evolution, phylogenomic, genome skimming, Urticaceae

## Abstract

Urticeae *s.l.*, a tribe of Urticaceae well-known for their stinging trichomes, consists of more than 10 genera and approximately 220 species. Relationships within this tribe remain poorly known due to the limited molecular and taxonomic sampling in previous studies, and chloroplast genome (CP genome/plastome) evolution is still largely unaddressed. To address these concerns, we used genome skimming data—CP genome and nuclear ribosomal DNA (18S-ITS1-5.8S-ITS2-26S); 106 accessions—for the very first time to attempt resolving the recalcitrant relationships and to explore chloroplast structural evolution across the group. Furthermore, we assembled a taxon rich two-locus dataset of *trnL-F* spacer and ITS sequences across 291 accessions to complement our genome skimming dataset. We found that Urticeae plastomes exhibit the tetrad structure typical of angiosperms, with sizes ranging from 145 to 161 kb and encoding a set of 110–112 unique genes. The studied plastomes have also undergone several structural variations, including inverted repeat (IR) expansions and contractions, inversion of the *trnN-GUU* gene, losses of the *rps19* gene, and the *rpl2* intron, and the proliferation of multiple repeat types; 11 hypervariable regions were also identified. Our phylogenomic analyses largely resolved major relationships across tribe Urticeae, supporting the monophyly of the tribe and most of its genera except for *Laportea*, *Urera*, and *Urtica*, which were recovered as polyphyletic with strong support. Our analyses also resolved with strong support several previously contentious branches: (1) *Girardinia* as a sister to the *Dendrocnide*-*Discocnide*-*Laportea*-*Nanocnide*-*Zhengyia*-*Urtica*-*Hesperocnide* clade and (2) *Poikilospermum* as sister to the recently transcribed *Urera sensu stricto*. Analyses of the taxon-rich, two-locus dataset showed lower support but was largely congruent with results from the CP genome and nuclear ribosomal DNA dataset. Collectively, our study highlights the power of genome skimming data to ameliorate phylogenetic resolution and provides new insights into phylogenetic relationships and chloroplast structural evolution in Urticeae.

## Introduction

Urticaceae, commonly known as the nettle family, is a cosmopolitan group of plants comprising approximately 54 genera and ∼2,600 species circumscribed into six tribes (Boehmerieae Gaudich., Cecropiaceae Gaudich., Elatostemateae Gaudich., Forsskaoleae Gaudich., Parietarieae Gaudich., and Urticeae Lam. and DC.; Conn and Hadiah, 2009) with various distinct morphological characters (Stevens, 2017). For example, members of tribe Urticeae are well known for their stinging trichomes (Friis, 1993). Urticeae *sensu* Friis (1989) consists of 10 genera of vast economic importance as sources of fiber (Singh and Shrestha, 1988; Bodros and Baley, 2008; Gurung et al., 2012) medicine (Momo et al., 2006; Tanti et al., 2010; Luo et al., 2011; Benvenutti et al., 2020; Sharan Shrestha et al., 2020), and food (Di Virgilio et al., 2015; Mahlangeni et al., 2020). This generic circumscription of the Urticeae, however, was established prior to the era of molecular phylogenetics. With the advent of the molecular tools, classification within tribe Urticeae has received much attention, with both taxonomic and phylogenetic studies spurring realignments (Hadiah et al., 2008; Kim et al., 2015; Huang et al., 2019; Wells et al., 2021). Molecular analyses have led to the exclusion of *Gyrotaenia* and the inclusion of *Touchardia*, *Poikilospermum* and *Zhengyia* in the tribe; hence, Urticeae presently comprises 12 genera (Wu et al., 2013; Kim et al., 2015; Jin et al., 2019). Molecular phylogenetic studies have also been able to demonstrate the monophyly of this tribe as well as which genera are polyphyletic or monophyletic.

**FIGURE 1:**
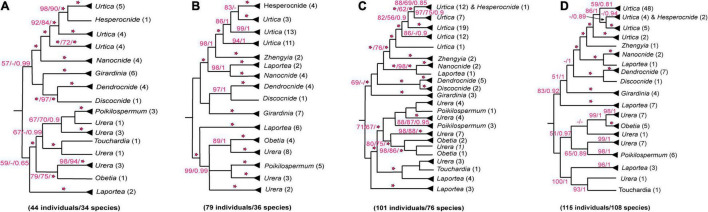
Alternative relationships of Urticeae based on combined loci data from previous *analyses*. **(A)** Wu et al. (2013): Maximum likelihood (ML)/Maximum parsimony (MP)/Bayesian inference (BI) analyses based on nuclear, chloroplast, and mitochondrial loci; **(B)** Kim et al. (2015): MP/BI tree inferred from chloroplast and nuclear DNA data; **(C)** Wu et al. (2018): ML/MP/BI tree inferred from nuclear, chloroplast, and mitochondrial data; **(D)** Huang et al. (2019): MP/BI *analyses* based on chloroplast and nuclear data. Numeric values besides each genus correspond to the total number of accessions sampled per genus and the number below each figure represents the total number (individual/species) of Urticeae samples used in each respective analysis. “*” indicates full support; “–“indicates no support in **(A,C)**, support values of < 75 (MP) and < 0.95 (BI) in **(B)**, and support values of < 50% (MP) and < 0.7 (BI) in **(D)**.

Although our understanding of evolutionary relationships of the tribe Urticeae has improved in recent years, some important nodes remain unresolved. For example, the phylogenetic position of *Laportea* remains contentious in previous studies. Wu et al. (2013), using seven combined markers from the mitochondrial, nuclear, and chloroplast genomes, recovered *Laportea* sister to a clade comprising *Obetia*-*Urera*-*Touchardia* and *Poikilospermum*, though with weak support (Figure 1A). Subsequent studies, however, have supported alternative, conflicting resolutions of *Laportea* (Figures 1B–D; Kim et al., 2015; Wu et al., 2018; Huang et al., 2019) probably due to the limited sampling. The placement of *Poikilospermum* also remains uncertain; although it has consistently been placed sister to *Urera*, support for this was either lacking (Figures 1A–C; Wu et al., 2013, 2018; Kim et al., 2015; Wells et al., 2021) or low (Figure 1D; Huang et al., 2019). The genus *Hesperocnide*, although supported as monophyletic in earlier studies, was recently recovered as polyphyletic by Huang et al. (2019), suggesting that further investigation of this genus may be required. Conflict concerning the placement of *Girardinia* further compounds taxonomic problems within Urticeae; several studies support its relationship with *Dendrocnide-Discocnide*, but without support (Figures 1A,B; Wu et al., 2013; Kim et al., 2015), while others (Wu et al., 2018; Huang et al., 2019) have recovered *Girardinia* sister to a clade comprising *Dendrocnide*-*Discocnide*-*Laportea*-*Nanocnide*-*Zhengyia*-*Urtica*-*Hesperocnide*, albeit also with low support (Figures 1C,D). These uncertainties around phylogenetic relationships within Urticeae are likely due to limited taxon or genic sampling in previous studies. Therefore, a broadly sampled phylogenomic study should offer useful framework for resolving these outstanding problems and guiding revised taxonomic treatments of the tribe.

Chloroplasts are ubiquitous organelles in plants with tractable attributes that make them highly suitable for use in phylogenetic and phylogeographic studies (Demenou et al., 2020; Silverio et al., 2021; Simmonds et al., 2021; Wang et al., 2021). In Urticaceae, whole chloroplast genomes have proven to be indispensable for sequence variation exploration (Wang et al., 2020b; Li et al., 2021). More broadly, studies of chloroplast genomes have been useful for understanding molecular evolutionary patterns of gene duplication, loss, rearrangement, and transfer across angiosperms (Yan et al., 2018; Do et al., 2020; Liu et al., 2020a; Oyebanji et al., 2020), though discordant relationships may be caused by plastid capture and other evolutionary processes.

For the present study, we sequenced and examined chloroplast genomes (CP genome/plastome) of the tribe Urticeae in order to explore plastome structural evolution in the tribe and to reconstruct the first-ever full plastome phylogeny for the tribe. Furthermore, we generated a robustly sampled dataset of Urticeae (comprising 291 accessions) aimed at reconstructing a more taxonomically rich phylogeny for the tribe. Specifically, we aimed to (1) characterize structural changes in Urticeae plastomes, (2) resolve deep relationships in the tribe using different data partitioning strategies, and (3) evaluate and update existing classifications for Urticeae in the light of our phylogenetic results based on both plastome and nuclear data.

## Materials and Methods

### Taxon Sampling

In this study, we sampled a total of 106 accessions, comprising 90 ingroup accessions (58 spp. in 12 genera) from the tribe Urticeae, plus 12 accessions (12 spp. in 11 genera) from other Urticaceae tribes and four (3 spp. in 3 genera) from outside the family as outgroups. These represent the genome skimming—CP genome and the nuclear ribosomal DNA (18S-ITS1-5.8S-ITS2-26S) dataset for the phylogenetic analyses (Supplementary Table 1). Of the 106 accessions, 57 representative accessions (each a different taxon) were selected for CP genome structural analyses. To produce a more comprehensive phylogenetic framework for the tribe Urticeae, we also generated a new two-locus dataset of 291 accessions (145 spp. in 26 genera) based on ITS and the *trnL-F* intergenic spacer. The ITS and the *trnL-F* intergenic spacer dataset was sampled based on maximum taxon data availability on NCBI database. Of the 291 accessions included, 187 sequences were obtained from NCBI GenBank while the remaining were newly sequenced for this study. Information on the plant material (collection localities and voucher specimen numbers) and the associated GenBank accessions are listed in Supplementary Table 1.

### DNA Extraction and Sequencing

A modified cetyl trimethyl ammonium bromide (CTAB) protocol (Doyle and Doyle, 1987) was used to extract total DNA from both silica gel-dried leaves and herbarium samples. Genomic DNA from each sample was then assessed for quality and quantity using both a NanoDrop 2,000 spectrophotometer (Thermo Fisher Scientific, United States) and agarose gel electrophoresis before library preparation. The library was built using the NEBNext Ultra II DNA Library Prep Kit for Illumina (New England BioLabs) according to the manufacturer’s instructions. Sequencing was then done using the Illumina HiSeq X Ten platform, yielding 150 bp paired-end reads. For each individual, 2–4 Gb of clean data was generated.

### Assembly and Annotation

SPAdes (Bankevich et al., 2012) was used for *de novo* assembly of all sequences using kmer length of 85–111 bp. For the CP genome, we visualized and filtered the newly assembled contigs to generate a complete circular genome in both Bandage v. 0.80 (Wick et al., 2015) and Geneious v. 8.1 (Kearse et al., 2012). The newly assembled sequences were annotated using the reference genome *Debregeasia longifolia_*MBD01 (MN18994) in the Plant Genome Annotation (PGA) platform (Qu et al., 2019), followed by manual curation of genes in Geneious to check if the start and stop codons are correct. Furthermore, for CP genomes, tRNAscan-SE v. 1.21 (Schattner et al., 2005) was used to further verify the tRNA genes with default settings. We used Chloroplot (Zheng et al., 2020) to generate the physical maps of the CP genomes.

### Plastome Structural Variation Analyses

#### Patterns of Inverted Repeat Boundary Shifts and Inversion

We characterized the genomic features of the 57 unique plastomes, including their size, structure (SC and IR regions), protein coding (PCG) and other (tRNA and rRNA) genes, and GC content. The junctions between the IR and single copy (SC) regions were then compared and analyzed using Geneious v. 8.1 (Kearse et al., 2012). ProgressiveMAUVE (Darling et al., 2010) was used to detect gene rearrangements and inversions among Urticeae taxa with *Elatostema parvum* as the reference genome. Default settings were used in ProgressiveMAUVE to automatically calculate the seed weight (15), and calculate Locally Collinear Blocks (LCBs) with a minimum LCB score of 30,000.

#### Repeat Sequence Analyses

We searched for the occurrence and distribution of three types of repeats within the studied plastomes of the tribe Urticeae. First, the program REPuter (Kurtz et al., 2001) was used to identify dispersed repeat sequences (forward, reverse, complement, and palindromic) using the following constraint values: a hamming distance of 3, minimum repeat size of 30 bp, and a maximum computed repeat of 100. Second, the tandem repeats were identified using the online program Tandem Repeats Finder (Benson, 1999) with the alignment parameters match, mismatch, and indels set to 2, 7, and 7, respectively. For this analysis, the maximum period size and TR array size were limited to 500 and 2,000,000 bp, respectively, and the minimum alignment score for reporting repeats was set at 50. Third, we used a Perl-based microsatellite identification tool (MISA; Thiel et al., 2003) to search for simple sequence repeats (SSRs) (i.e., mono-, di-, tri-, tetra-, penta-, and hexanucleotide repeats) within Urticeae plastomes. The threshold values for this analysis were set at 10, 6, 5, 5, 5, and 5 for mono-, di-, tri-, tetra-, penta- and hexanucleotides, respectively.

#### Sequence Divergence Analyses

To illustrate interspecific sequence variation and gene organization of the entire plastomes across the 57 examined species, we used mVISTA with the shuffle-LAGAN mode (Frazer et al., 2004) and *E. parvum* as the reference genome. For the assessment of sequence divergence and exploration of highly variable chloroplast markers, a sliding window analysis was performed in DnaSP v. 6 (Rozas et al., 2017) to compute the nucleotide diversity (π) for all protein-coding (CDS) and non-coding (nCDS i.e., intron and intergenic spacer) regions. The step size was set to 300 bp, with a window length of 1,000 bp. The gene recovered to have the highest nucleotide diversity was then used to draw a phylogenetic tree to test the resolution of the identified barcode for our species.

### Phylogenetic Inference

Phylogenetic analyses were conducted using different partitioning schemes from two datasets: the genome skimming [CP genome and the 18S-ITS1-5.8S-ITS2-26S (nrDNA) sequences] and two-locus (ITS and the *trnL-F* intergenic spacer) dataset. We extracted the coding (CDS) and non-coding (nCDS) regions from the CP genome to elucidate the phylogenetic utility of the different regions. This partitioning is important as both CDS and nCDS regions have been shown to exhibit distinct rates of nucleotide substitution (Wolfe et al., 1987; Jansen and Ruhlman, 2012). In total, six molecular data matrices were generated to explore the phylogenetic relationships of the tribe Urticeae, of which five were from the genome skimming dataset: (1) Whole chloroplast (CP) genomes, (2) CP coding regions (CDS), (3) CP non-coding regions (nCDS), (4) nuclear ribosomal DNA (nrDNA), and (5) combined whole CP genomes and nuclear ribosomal DNA (CP + nrDNA). The final matrix (6) sampled the two-locus dataset *trnL-F* intergenic spacer and ITS sequences (*trnL-F* + ITS) across expanded taxonomic sampling of 291 accessions.

Phylogenetic analyses were conducted using maximum likelihood (ML) and Bayesian inference (BI) methods in RAxML v. 8.2.11 (Stamatakis, 2014) and MrBayes v. 3.2 (Ronquist et al., 2012), respectively. Substitution models for all the datasets were first determined based on Akaike information criterion (AIC; Akaike, 1973) in jModelTest2 v. 2.1.7 (Darriba et al., 2012; Supplementary Table 2). Maximum likelihood analyses was done in RAxML using the bootstrap option of 1,000 replicates. For BI analyses, we performed two independent runs, each consisting of four Markov Chain Monte Carlo (MCMC) chains, and sampling of one tree every 1,000 generations for 1 million (CP, nCDS, and CP + nrDNA), 3 million (CDS), and 20 million (*trnL-F* + ITS and only nrDNA) generations. The convergence of the MCMC chains of each run was determined when the average standard deviation of split frequencies (ASDSF) achieved ≤ 0.01, and adequate mixing was based on the Effective Sample Sizes (ESS) values ≥ 200. Stationarity was assessed by checking if the plot of log-likelihood scores had plateaued in Tracer v1.7.1 (Rambaut et al., 2018). The first 25% of the sampled trees acquired from all the runs were discarded as burn-in, and consensus trees were constructed from the remaining trees to estimate posterior probabilities.

## Results

### Chloroplast Genome Organization

**FIGURE 2:**
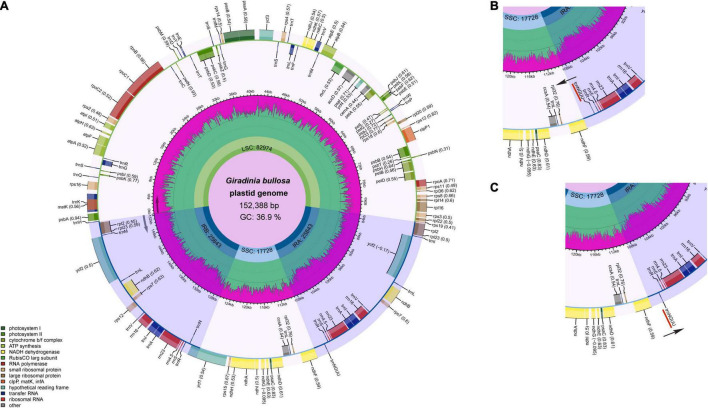
**(A)** Gene map of complete chloroplast genome of *Girardinia bullosa* (a typical representative of gene organization in Urticeae *s.l.* plastomes); **(B)** inset map showing the inverted orientation of *trnN-GUU* in clade 3C except for *Discocnide mexicana*; **(C)** inset map of the Urticeae plastome, showing the typical orientation of trnN-GUU. Genes inside and outside the outer circle are transcribed clockwise and counterclockwise, respectively.

The plastomes of Urticeae species varied greatly in sequence length, ranging in size from 145,419 bp (*Nanocnide lobata*) to 161,930 bp (*Laportea grossa*) (Table 1). All exhibited a quadripartite structure typical of angiosperms (Figure 2A)—a pair of IRs (24,593–30,335 bp) separated by the LSC (77,955–84,521 bp) and SSC regions (16,500–19,838 bp). We observed a marginal difference in the GC content across the whole plastome (36.3–37.2%) and its elements — the IR (41.8–43.3%), LSC (33.8–34.7%), and SSC (29.6–31.1%) regions.

**TABLE 1:** Summary of sizes of the whole Urticeae plastomes, and the three compartments.

Species	Nucleotide length (bp)			
	Genome	LSC	SSC	IR
*Dendrocnide basirotunda*_J2078	152,646	83,433	18,229	25,492
*Dendrocnide meyenia*_D7	152,621	83,430	18,149	25,521
*Dendrocnide sinuata*_J7885	152,559	83,348	18,187	25,512
*Dendrocnide urentissima*_D4	152,658	83,444	18,230	25,492
*Discocnide mexicana*_W268	153,327	83,841	17,580	25,953
*Girardinia bullosa*_A1	152,388	82,974	17,728	25,833
*Girardinia chingiana*_G1	152,659	83,451	18,068	25,570
*Girardinia diversifolia*_G56	152,979	83,636	18,127	25,608
*Girardinia formosana hayata*_G3	152,596	83,364	18,056	25,588
*Girardinia suborbiculata* subsp. *grammata*_G22	152,687	83,453	18,020	25,607
*Girardinia suborbiculata* subsp. *suborbiculata*_G15	152,894	83,650	18,104	25,570
*Girardinia suborbiculata* subsp. *triloba*_G19	152,874	83,516	18,142	25,608
*Hesperocnide tenella*_W61	146,864	79,555	17,691	24,809
*Laportea aestuans*_L30	153,521	82,883	16,500	27,609
*Laportea bulbifera*_GLGE14842	149,436	81,759	17,859	24,909
*Laportea canadensis*_W167	150,253	82,394	17,783	25,038
*Laportea cuspidata*_L27	149,149	80,905	17,450	25,397
*Laportea decumana*_L15	151,855	82,777	18,080	25,499
*Laportea grossa*_L2	161,930	83,658	19,838	29,217
*Laportea medogensis*_GLGE141037	150,196	82,385	17,759	25,026
*Laportea mooreana*_L12	150,827	81,878	18,371	25,289
*Laportea ovalifolia*_L14	153,659	82,193	16,596	27,435
*Nanocnide japonica*_N3	145,970	78,396	17,300	25,137
*Nanocnide lobata*_N6	145,419	77,955	17,258	25,103
*Obetia aldabrensis*_W291	153,239	84,219	18,628	25,196
*Poikilospermum cordifolium*_Poi7	153,801	84,436	18,617	25,374
*Poikilospermum lanceolatum*_Poi8	153,879	84,521	18,618	25,370
*Poikilospermum naucleiflorum*_Poi6	153,782	84,414	18,600	25,384
*Touchardia latifolia*_T2	152,871	84,003	18,252	25,308
*Urera baccifera*_Ur21	153,215	84,314	18,027	25,437
*Urera cameroonensis*_Ur12	153,212	83,990	18,532	25,345
*Urera capitata*_W143	153,771	84,297	18,626	25,424
*Urera* cf *cordifolia*_Ur15	153,214	83,992	18,536	25,343
*Urera glabra*_Ur17	152,663	83,499	18,502	25,331
*Urera hypselodendron*_Ur16	153,212	84,007	18,515	25,345
*Urera oligoloba*_Ur23	153,919	84,056	18,561	25,151
*Urera robusta*_Ur19	153,198	84,017	18,491	25,345
*Urtica angustifolia*_J3303	146,703	79,830	17,683	24,595
*Urtica ardens*_GLGE152058	146,795	79,693	17,686	24,708
*Urtica atrichocaulis*_S11193	146,717	79,884	17,633	24,600
*Urtica chamaedryoides*_W162	146,455	79,304	17,701	24,725
*Urtica dioica* subsp. *xijiangensis*_U41	147,935	79,627	17,530	25,389
*Urtica dioica*_W174	146,928	80,052	17,676	24,600
*Urtica domingensis*_W145	146,125	79,260	17,665	24,600
*Urtica hyperborea*_J5455	147,898	79,748	17,588	25,281
*Urtica kioviensis*_U24	146,725	79,855	17,666	24,602
*Urtica macrorrhiza*_U50	146,747	79,886	17,661	24,600
*Urtica magellanica*_U33	146,606	79,613	17,657	24,668
*Urtica mairei*_J1664	146,790	79,689	17,685	24,708
*Urtica membranifolia*_S13031	158,078	79,719	17,689	30,335
*Urtica morifolia*_U200	146,755	79,643	17,690	24,711
*Urtica radicans*_U21	146,667	79,819	17,662	24,593
*Urtica rupestris*_U28	146,751	79,859	17,696	24,601
*Urtica* sp_U19	147,508	79,069	17,669	25,385
*Urtica thunbergiana*_J2498	146,846	79,667	17,711	24,734
*Urtica urens*_W175	147,516	79,076	17,668	25,386
*Zhengyia shennongensis*_Zh1	150,109	81,186	17,885	25,519

*LSC, Large Single Copy; SSC, Small Single Copy; IR, Inverted Repeat.*

A range of 110–112 unique genes was detected across these plastomes, including 76–78 PCGs, 30 tRNA genes, and 4 rRNA genes. The IR region had complete duplications for 7 tRNA genes, 6 PCGs, and 4 rRNA genes. Across all 57 plastomes, 15 genes had a single intron (*atpF, ndhA, ndhB*, *petB*, *petD*, *rpl2*, *rpl16*, *rpoC1*, *rps16*, *trnA-UGC, trnG-UCC*, *trnI-GAU*, *trnK-UUU*, *trnL-UAA*, and *trnV-UAC*), while two genes (*clpP* and *ycf3*) had two introns. The *rps12* gene was spliced into two transcriptions, with one exon in the LSC and two in the IR. Notably, the *rpl2* gene of *Hesperocnide tenella* and most *Urtica* taxa except for *Urtica dioica* subsp. *xijiangensis*_U41, *Urtica dioica*_J5488, *Urtica hyperborea*_J5455, *Urtica* sp_U19, and *Urtica urens* lacked an intron. Apart from the region containing an inverted *trnN-GUU* in five species (four *Dendrocnide* species and *Laportea decumana*; Figures 2B,C), no significant gene rearrangement was observed within the studied plastomes (Supplementary Figure 1A).

### Inverted Repeat Expansion and Contraction

**FIGURE 3:**
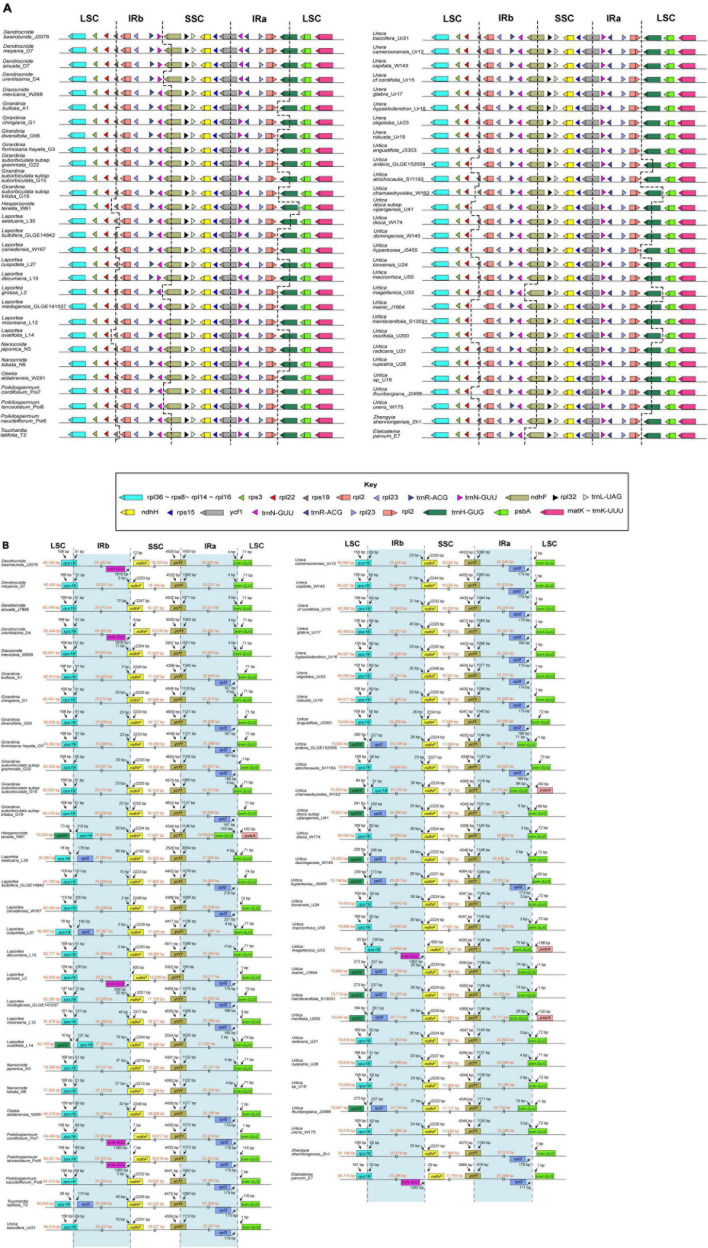
**(A)** representative map showing expansions and contractions in the IR region; **(B)** comparison of the IR/SC junctions among 57 Urticeae plastomes. The genes around the borders are shown above or below the main line. LSC, Large Single Copy; SSC, Small Single Copy; IR (a and b), Inverted Repeat a and b.

Comparison of the IR boundaries among the 57 plastomes from tribe Urticeae revealed varying expansion and contraction of the IRs (Figure 3A). Herein, we report only the functional genes located at the IR-SC boundaries. The LSC/IRb border was embedded in the *rps19* gene (with 50–131 bp located within IRb) in 43 taxa. The remaining 14 species showed: an expansion in three species (*rpl22* in the LSC—*rps19* in the IRb); contraction (*rps*19 in the LSC—*rpl2* in the IRb) of the IR in three species; the loss of the *rps19* gene in eight species (*rpl22* in the LSC—*rpl2* in the IRb), causing variations in the boundary (Figure 3B). The IRb/SSC boundary generally fell within the *ndhF* gene (with 50–131 bp located at IRb), except in six species where the boundary was detected in the intergenic region of *trnNGUU*-*ndhF* (Figure 3B). We observed that the IRa/LSC boundary of most species lay within either the intergenic *rpl2*-*trnHGUG* or non-coding *trnH-GUG* regions, except for four species (*Hesperocnide tenella*_W61, *Urtica chamaedryoides*_W162, *Urtica magellanica*_U33, and *Urtica morifolia*_U200) in which the boundary was located within the intergenic region *trnH-GUG*—*psbA* (Figure 3B). The most conserved boundary across species was that of the SSC/IRa, which was always positioned within the *ycf1* coding gene, which had a length of 195–3,054 bp overlapping into the IRa region (Figure 3B).

### Repeat Structure and Search for Simple Sequence Repeats

**FIGURE 4:**
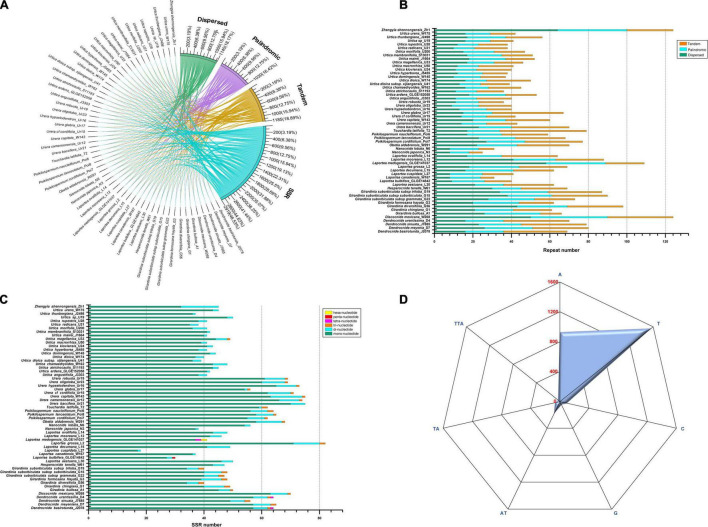
**(A)** Chord diagram showing a connection between species and their corresponding repeat types (Dispersed, Palindromic, Tandem, and SSR). The tick marks beside each repeat type indicate the frequency of the number of repeats detected and their percentages, respectively; **(B)** frequency of tandem, palindromic, and dispersed repeats; **(C)** number of the six SSR(simple sequence repeats) nucleotides; **(D)** the seven most abundant SSR motifs in Urticeae plastomes. The values along the axis represent the abundance values.

The 57 Urticeae plastomes showed a total of 6,274 repeats based on four classifications (Figure 4A and Supplementary Table 3). Generally, the most frequent repeat type was the SSR (2,919, 46.53%), followed by tandem (1,185, 18.89%), dispersed (1,140, 18.17%), and palindromic repeats (1,030, 16.42%) (Figure 4A). The distribution of the dispersed, tandem, and palindromic repeats varied between 25 (*Nanocnide japonica*_N3) and 124 (*Discocnide mexicana*_W268 and *Zhengyia shennongensis*_Zh1) (Figure 4B), and that of the number of SSRs ranged from 18 (*Laportea cuspidata*_L27) to 82 (*Laportea grossa*_L2) (Figure 4C). The majority of the SSRs were mononucleotides (2,627, 89.97%), with poly-A and poly-T SSR motifs being the two most frequent (Figure 4D and Supplementary Table 5). Dinucleotides, trinucleotides, tetranucleotides, pentanucleotides, and hexanucleotides accounted for 8.50, 1.27, 0.14, 0.03, and 0.07% of the SSR repeats, respectively (Figure 4C and Supplementary Table 4).

### Sequence Divergence Analysis

Pairwise comparison of divergent regions within the 57 Urticeae plastomes using mVISTA revealed very low intra- and inter-generic (Supplementary Figure 1B) sequence divergence across the plastomes. Moreover, nCDS regions were generally more divergent and had higher levels of variation than CDS regions (Supplementary Figures 1B, 2). For the CDS, the top five genes with the highest nucleotide diversity (π) values (all with π > 5%) were *rpoc2*, *cemA*, *rpoA*, *rpl22*, *ccsA*, and *ycf1* (Supplementary Figure 2A). The most variable nCDS regions were the *trnQ(UUG)*—*psbK*, *trnG(GCC)*—*trnfM(CAU)*, *ycf3*—*trnS(GGA)*, *cemA*—*petA*, and *ndhE*—*ndhG* spacer regions, all with π > 10% (Supplementary Figure 2B). The *ycf*1 gene tree depicted highly resolved and supported relationships, owing to the gene’s high nucleotide diversity (Supplementary Figure 2C).

### Phylogenetic Relationships

The sequence characteristics, tree diagnostic values, and the best-fit model determined by jModelTest for all datasets are given in Supplementary Table 2. The phylogenetic results presented here are based on both ML and BI analyses. The ML and BI analyses generated here generally had nearly identical topologies with few differences at the shallow nodes. Factors driving discrepancies between the ML and BI topologies have been previously reported (Huelsenbeck, 1995; Sullivan and Joyce, 2005; Som, 2014). Of those, the optimality criterion and specific hypotheses in the modeling of sequence evolution are parsimonious to explain the few discrepancies between the ML and BI topologies inferred from the same data matrix in our study. In most cases, the phylogenetic relationships inferred from ML were discussed because it has the most supporting shreds of evidence from the morphological affinities between the known species within the tribe Urticeae. The phylogenetic relationships constructed for each data matrix are further reported.

### Chloroplast Data Analyses

The CDS, nCDS, and whole CP phylogenetic trees were largely identical in their topologies with only a few exceptions concerning the relationships of two clades 3F3I and 3F3II (Supplementary Figures 3A–CI). In the CDS data, these were sister to one another, hence formed a monophyletic clade 3F3 (Supplementary Figure 3A). However, in the whole CP dataset, 3F3I was sister to both 3F3II, and 3F4, while in nCDS dataset, 3F3II was sister to both 3F3I and 3F4 (Supplementary Figures 3B,CI). Nevertheless, it should be noted that the whole CP dataset generally had better support compared to both the CDS and nCDS datasets.

### nrDNA Data Analysis

Regarding relationships between major clades in Urticeae, the results from the nrDNA dataset (Supplementary Figure 3CII) recovered almost congruent relationships with that of the whole CP dataset (Supplementary Figure 3CI), other than a few discrepancies in particular major clades and phylogenetic placement of some species. For instance, in the nrDNA phylogeny, clade 3D (*Girardinia*) was recovered as sister to clade 3C (Supplementary Figure 3CII), whereas in whole CP phylogeny, clade 3D was recovered as sister to a clade comprising subclades 3C, 3B, and 3A (Supplementary Figure 3CI). The sister relationships of clade 3G, and those within clade 3E-F also changed depending on the dataset examined. Moreover, we found slight differences in some shallower relationships between the whole CP and nrDNA phylogenies (e.g., the contradicting phylogenetic positions of *Dendrocnide urentissima*, *Girardinia suborbiculata* subsp. *suborbiculata*, etc.; Supplementary Figure 3C). These differences were, however, mostly restricted to areas of poor support, and the whole CP phylogeny was generally better supported than that of nrDNA.

### Combined Whole Chloroplast Genome and nrDNA (CP + nrDNA) Analysis

**FIGURE 5:**
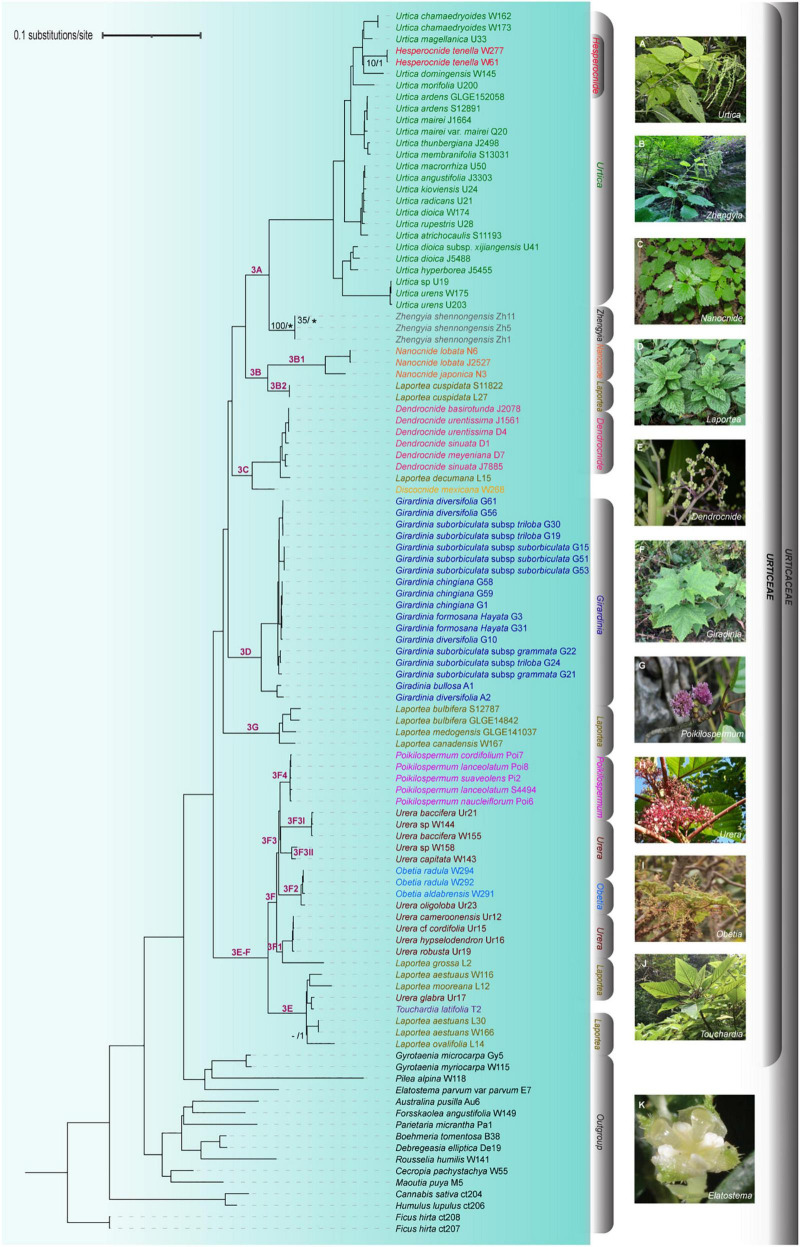
Phylogenetic relationships of Urticeae inferred from maximum likelihood (ML) and Bayesian inference (BI) based on combined complete plastome and nrDNA sequences. Numbers on the branch indicate clade classification (in purple) and ML_BS/BI_PP values (in black)—note that branches with no support values indicate both ML_BS ≥ 90 and BI_PP = 1.00; lastly, “*” indicate incongruence between ML and BI trees and “–” no support values. Representative images of genera within Urticeae *s.l.* are shown on the right. Photographs: **(A–C,E,G,K)** by Z.Y. Wu, **(D,F)** by C.A. Ogoma, **(H)** by U. Dreschel, **(I)** by C. Kunath, and **(J)** photographed by J. Cantley.

Phylogenetic resolution and node support values were significantly improved by the combination of whole CP genome and nrDNA data (Figure 5). The phylogeny inferred from the combined data matrix was the best resolved and supported phylogenetic tree amongst all the other data matrices, and was more similar in topology to the three chloroplast data matrices (whole CP, CDS, and nCDS, regions) than the nrDNA one (Figure 5 and Supplementary Figures 3A–C). The monophyly of Urticeae was strongly supported (BS/PP = 100/1), with Elatostemeae as its sister tribe (Figure 5). Generally, the phylogeny was well resolved, with most nodes being strongly supported by both ML and BI analyses, except the placement of *Zhengyia shennongensis* (BS = 100 PP = “–“), the relationship between *Urtica domingensis* and *Hesperocnide tenella* (BS = “–“ PP = 1), and the relationship between *Laportea aestuans* and *Laportea ovalifolia* (BS = “–“ PP = 1) (Figure 5). Nine genera within Urticeae were recovered as monophyletic (*Dendrocnide*, *Discocnide*, *Girardinia*, *Hesperocnide, Obetia*, *Nanocnide*, *Poikilospermum*, *Touchardia*, and *Zhengyia*) and three as polyphyletic (*Urtica*, *Laportea*, and *Urera*), all with strong support. For ease of discussion, we sectioned Urticeae into six major clades, each with full bootstrap support; the names reflect the clade naming system of Wu et al. (2013). They include Clade 3A (*Urtica*, *Hesperocnide*, and *Zhengyia*), Clade 3B (*Nanocnide* and *Laportea cuspidata*), Clade 3C (*Dendrocnide*, *Discocnide*, and *Laportea decumana*), Clade 3D (*Girardinia*), and Clade 3G (*Laportea*). Clade 3E-F was recovered as sister to the rest of the Urticeae tribe with maximum support, and comprised *Poikilospermum*, *Urera*, *Obetia*, and *Laportea*. Within it, *Poikilospermum* (sub-clade 3F4) was recovered for the first time as a sister clade to *Urera* (sub-clade 3F3) with full support (Figure 5). *Urera* comprised three separate subclades within Clade 3E-F, each with strong support. Moreover, in this study *Laportea* was split into five different clades. Clade 3D (*Girardinia*) was also recovered for the first time as sister to a clade comprising 3A, 3B, and 3C, with full support.

### Combined Analysis of *trnL-F* + ITS

**FIGURE 6:**
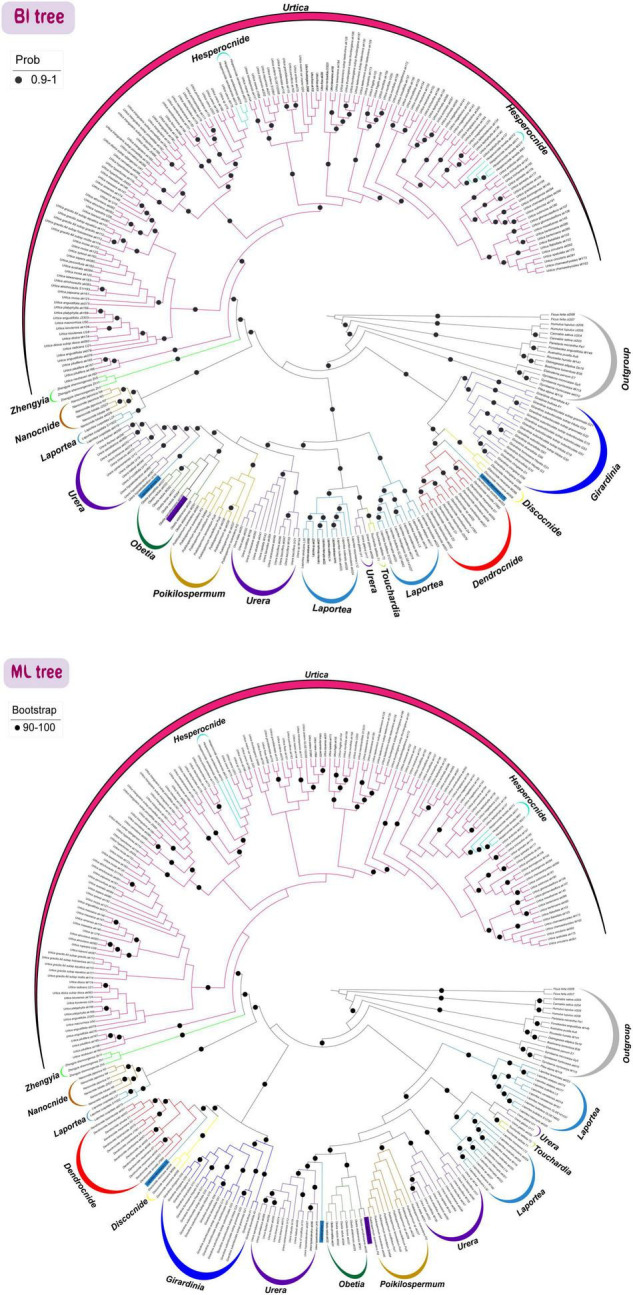
Phylogenetic relationships of Urticeae tribe inferred from maximum likelihood (ML) and Bayesian inference (BI) based on *trnL-F* intergenic spacer and ITS (*trnL-F* + ITS) loci sequences. Support values indicated by black circle show ML_BS ≥ 90 and BI_PP ≥ 0.90.

The tree topology from the analysis of the *trnL-F* and ITS dataset was largely congruent with the previously published phylogenies inferred from a small number of loci. Eight genera were strongly supported as monophyletic (i.e., *Dendrocnide*, *Discocnide*, *Girardinia*, *Obetia*, *Nanocnide*, *Poikilospermum*, *Touchardia*, and *Zhengyia*) while four genera were recovered as polyphyletic (i.e., *Hesperocnide*, *Urtica*, *Laportea*, and *Urera)*. *Hesperocnide* was recovered here as polyphyletic (BS/PP > 90/0.90 and BS/PP < 90/0.90; Figure 6) as compared to the combined whole (CP + nrDNA) where it was retrieved as monophyletic with full bootstrap support (Figure 5). Moreover, most of the shallow nodes of *trnL-F* and *ITS* tree received lower bootstrap support (Figure 6) compared to the combined whole (CP + nrDNA) tree, in which nearly all the nodes were fully supported.

## Discussion

### Plastome Structural Evolution

All 57 Urticeae CP genomes examined are quadripartite but varied in size. The observed range was consistent with chloroplast genome sizes of angiosperms (Zhang et al., 2021) and the few existing sequenced plastomes of Urticaceae (Wang et al., 2020b; Li et al., 2021), which range between 120 and 180 kb. Of the plastomes in our study, *Laportea grossa* had the largest genome, while *Nanocnide lobata* had the smallest, implying that CP genomes in Urticaceae are structurally different. Also, the number of PCGs in the Urticeae plastomes in our study (76–78) was comparable with the typical range for angiosperm plastomes (70–88 genes) (Wicke et al., 2011). Likewise, we found congruence with the range of GC content previously reported in other plastomes of Urticaceae, e.g., *Pilea mollis* (36.72%; Li et al., 2021), *Elatostema dissectum* (36.2%; Fu et al., 2019), *Droguetia iners* (36.9%), and *Debregeasia elliptica* (36.4%) (Wang et al., 2020b). Generally, the GC content had no significant phylogenetic implication in our study. Moreover, consistent with previous studies (Li et al., 2020, 2021; Dong et al., 2021), the GC content was higher in the IR than in the SC. The GC inequality perhaps also plays a significant factor in the conservatism of the IR region compared to the SC regions (Li et al., 2020).

Among the genes present in our Urticeae plastomes, *rpl2* was noteworthy, considering that 18 of the examined species had no introns for this gene. Intron loss has been widely documented in angiosperm plastomes: e.g., *Avena sativa* (*rpoC1* intron loss; Liu et al., 2020b), *Cicer arietinum* (*rps12* and *clpP* intron losses; Jansen et al., 2008), *Lagerstroemia* (*rpl2* intron loss; Gu et al., 2016), and Asteropeiaceae + Physenaceae (*rpl2* intron loss; Yao et al., 2019). Another notable structural change found here was an inversion of the *trnN-GUU* gene, which is a synapomorphy of the clade 3C, except for the clade’s basal species *Discocnide mexicana* (Figure 2B). Gene inversions have also been detected in many angiosperm plastomes, including those of Poaceae (Guisinger et al., 2010), Styracaceae (Yan et al., 2018), Orchidaceae (*Uncifera acuminata*; Liu et al., 2020a), and Adoxaceae (Wang et al., 2020a). The latter, involving the inversion of the *ndh*F gene in Adoxaceae, is relevant to our study since it involves only one gene that also borders the inverted gene in our study (*trnN-GUU*). Typically, plastome inversions are deemed highly valuable in phylogenetics owing to their relative rarity, easily determined homology, and easily inferred state polarity (Cosner et al., 1997; Dugas et al., 2015; Schwarz et al., 2015). Despite some significant research efforts regarding the intramolecular recombination between dispersed short inverted/direct repeats and tRNA genes (Cosner et al., 1997; Haberle et al., 2008; Sloan et al., 2014), the cause of inversions in plant genomes remains unclear.

Our analyses showed that IR expansion and contraction vary across Urticeae, and lack taxonomic utility at a broader scale. Mostly, the SC/IR borders are relatively conserved among angiosperm plastomes and usually located within the *rps19* or *ycf1*gene (Downie and Jansen, 2015), even though it is assumed that IR expansion or contraction is accompanied by the shift of genes located in the IR/SC boundary (Zhu et al., 2016). Similar IR/SC changes are also evident in other Urticaceae plastomes (Wang et al., 2020b; Li et al., 2021). Changes in the IR/SC junctions have been considered one of the main drivers of the size diversity in the CP genomes of higher plants (Ma et al., 2013; Yang et al., 2016; Yan et al., 2018; Xue et al., 2019). Notably, we found the loss of the *rps19* gene to be the most parsimonious explanation for the diversification of the genes bordering the IR/LSC in the eight plastomes examined from the genus *Urtica*—(*U. ardens*_GLGE152058, *U. dioica* subsp. *xijiangensis*_U41, *U. domingensis*_W145, *U. hyperborea*_J5455, *U. mairei*_J1664, *U. membranifolia*_S13031, *U. morifolia*_U200, and *U. thunbergiana*_J2498; Figure 3A).

We detected several repeat types within the sampled plastomes of tribe Urticeae, among which SSRs were the most frequent, accounting for 46.53% of the repeats (Figure 4A). The most abundant SSRs were mononucleotide homopolymers, particularly poly−A and T motifs (Figure 4D and Supplementary Table 5). This phenomenon of A/T motif abundance has also been reported in *Pilea* (Li et al., 2021) and *Debregeasia* (Wang et al., 2020b) species, and might occur because the A/T motifs are more frequently dynamic compared to G/C (Li et al., 2020). Generally, it is presumed that repeat sequences are closely connected with a vast number of indels; therefore, the more abundant they are, the greater the nucleotide diversity (McDonald et al., 2011). Hence, the chloroplast repeat sequences could be potential sources of variation for evolutionary studies, and population genetics (Xue et al., 2012). We also found higher nucleotide diversity in the nCDS than in the CDS regions, consistent with findings from other taxa (Jansen and Ruhlman, 2012; Huang et al., 2014). Although the nucleotide content of chloroplast genomes is usually relatively stable, with a highly conserved gene structure (Jansen et al., 2005; Ravi et al., 2008; Wicke et al., 2011), mutation hotspots still exist within it (Zhang et al., 2021). We detected a total of 11 hypervariable loci in both CDS and nCDS regions (Supplementary Figure 2) that could be potentially used as DNA barcodes in future studies of this group. Among them was the locus *ycf1*, which was also reported in previous Urticaceae studies (Wang et al., 2020b; Li et al., 2021) as a highly variable locus with great taxonomic utility. Moreover, a study by Dong et al. (2015) reinforces this view, and recommemnds y*cf*1 as a suitable plastid barcode for land plants. Indeed, our y*cf*1 phylogenetic tree (Supplementary Figure 2C) is consistent with the above studies, especially with regard to the high resolution and support level. Therefore, we suggest that *ycf1* represents a highly useful molecular marker, not just for tribe Urticeae, but likely for the entire family. Presently, DNA barcodes are widely used in species identification, resource management, and studies of phylogeny and evolution (Gregory, 2005; Liu et al., 2019).

### Phylogenetic Relationships of Urticeae

#### Phylogenetic Relationships Based on Genome Skimming (CP Genome + nrDNA) Data

The combined matrix (CP genome + nrDNA) yielded a well-supported phylogeny and resolved many relationships of the tribe Urticeae depite the topological difference in clades 3(D, 3G, and E-F), between the two separate datasets (Supplementary Figure 3C). This resolution shown by the combined matrix may be ascribed to the greater number of phylogenetically informative plastid sites (Supplementary Table 2). Moreover, it could be due to a weak phylogenetic signal in the nrDNA that agrees and complements the signal of the CP matrix. However, beyond some major conflicts, the individual CP and nrDNA trees are generally in agreement with most conflicting relationships pertaining to poorly supported areas of the phylogeny, although we did not perform follow-up analyses to identify what this means for different parts of the tree. Cases of topological dissimilarity are often reported in phylogenetic studies (Wendel and Doyle, 1998; reviewed by Degnan and Rosenberg, 2009). This phenomenon can be best explained by a number of factors including differences in taxon sampling, incomplete lineage sorting, hybridization/introgression, paralogy, gene duplication and/or loss, and horizontal gene transfer (Degnan and Rosenberg, 2006; Naciri and Linder, 2015; Lin et al., 2019; Nicola et al., 2019). Hence, as more samples become available, future studies should investigate the factors responsible for the observed conflicting relationships within the Urticeae.

Our study represents the first phylogeny of the tribe Urticeae based on a broad sampling of both CP genomes and nrDNA sequences. Importantly, we clarify which of the Urticeae genera are strongly supported as monophyletic or polyphyletic (Figure 5). Compared to previous studies based on a limited number of genes (Hadiah et al., 2008; Deng et al., 2013; Wu et al., 2013, 2018; Kim et al., 2015; Grosse-Veldmann et al., 2016; Huang et al., 2019; Wells et al., 2021), we exploited the utility of whole CP genomes for resolving phylogenetic relationships in Urticeae, and also revealed the most informative sites and regions across the plastome. Our results proved to be largely consistent with most of the recently established phylogenetic relationships of Urticeae based on a range of 3–7 selected marker regions (Wu et al., 2013, 2018; Kim et al., 2015; Huang et al., 2019; Wells et al., 2021). In general, however, our data improved resolution throughout Urticeae compared with previous studies, with almost all nodes being fully supported, especially those previously known to be problematic. Four of the most important new phylogenetic insights generated by the current study are discussed below.

First, the sister relationship of *Girardinia* has been contentious. *Girardinia* had been resolved as sister to *Dendrocnide-Discocnide* based on chloroplast, mitochondrial, and nuclear data (Wu et al., 2013), and using ITS, *rbcL*, and *trnL-F* regions (Kim et al., 2015), but without support in either case. Subsequently, using expanded taxon sampling and five markers from both the nuclear and CP genomes, the sister relationship of *Girardinia* to *Dendrocnide*-*Discocnide*-*Laportea*-*Nanocnide*-*Zhengyia*-*Urtica*-*Hesperocnide* was resolved, but with limited support (Wu et al., 2018; Huang et al., 2019). Our results support this latter relationship but with maximum support (BS/PP = 100/1), for the first time.

Second, our molecular phylogeny of the “*Urera* alliance clade” (this study clade 3E-F) corroborated the generic delimitation and subdivisions of the “*Urera* clade” from Wells et al. (2021), and showed two clades of *Laportea* (which they did not examine) as also a member (Figure 5). Their division of the paraphyletic *Urera* into three genera was strongly supported here: these were *Urera s.s.* (our Clade 3F3), *Scepocarpus* (entirely African; our clade 3F1, which also includes *Laportea grossa*), and an expanded *Touchardia* (part of clade 3E, that includes *Urera glabra* from Hawaii and three species of *Laportea* as per our study). Our data suggests that the two *Laportea* clades should hence be fully examined and considerations made as to whether to subsume them within the resurrected *Scepocarpus* and the expanded *Touchardia*.

Third, previous studies (Kim et al., 2015; Wu et al., 2018; Huang et al., 2019) have typically resolved *Laportea* into three clades. For instance, Kim et al. (2015) recovered three *Laportea* clades corresponding to sections *Laportea* Gaudich. (*L*. *alatipes*, *L*. *bulbifera*, *L*. *canadensis*, *L*. *lanceolata*), *Sceptrocnide* (Maxim.) C. J. Chen (*L*. *cuspidata*), and *Fleurya* (Gaudich.) Chew [*L*. *aestuans* (L.) Chew, *L*. *interrupta*, *L*. *ruderalis* (G. Forst.) Chew], consistent with the sectional classification of Wang and Chen (1995). Our analysis, however, resolved *Laportea* into five major clades. Moreover, we found that *L. aestuans* was polyphyletic: one subgroup was sister to *L. mooreana* with full support and the other was sister to *L. ovalifolia* with support of BS/PP = –/1. The latter relationship was detected by Wu et al. (2018) but without support. However, other studies found different relationships: *L. aestuans* as sister to *L. interrupta*, and *L. ruderalis* with full support according to Kim et al. (2015), or sister to *L. ruderalis* and *L. peduncularis* with support of MP/PP = 96/1 according to Huang et al. (2019). These discrepancies likely reflect differences in taxon and molecular sampling—with a wider sampling of populations, *L. aestuans* might comprise more than two unrelated clades. While additional study on *Laportea* is clearly needed, the current study provides one of the most comprehensive phylogenetic perspectives on this little-studied genus. Future investigations should, however, employ more extensive molecular data across the entire phylogenetic spectrum of *Laportea* to further clarify its relationships and the number of lineages.

Finally, our analysis resolved the sister relationship between *Poikilospermum* and *Urera* previously obtained by Huang et al. (2019), but replacing their modest support (BS/PP = 65/0.89) with full support (BS/PP = 100/1) for the first time.

#### Comparison Between Genome Skimming (CP Genome + nrDNA) and Two-Locus (*trnL-F* + ITS) Phylogeny

In our study, the trees inferred from both the CP genome + DNA and the two-locus dataset (*trnL-F* + ITS) provided full support for the monophyly of Urticeae. However, the CP genome + nrDNA tree presented a higher percentage of fully supported nodes compared with that of the two-locus tree (Figures 5, 6). This underscores the importance of genome-scale datasets for resolving major recalcitrant relationships.

The most notable finding from our two-locus phylogenetic analysis was the reconstruction of *Hesperocnide* as polyphyletic, consistent with Huang et al. (2019). Our current CP genome + nrDNA analysis and prior molecular studies, however, recovered *Hesperocnide* as monophyletic (Kim et al., 2015), with a close relationship to *Urtica* (Sytsma et al., 2002; Hadiah et al., 2008; Deng et al., 2013; Wu et al., 2013; Kim et al., 2015). The polyphyletic results from the two-locus tree can be ascribed to the sampling of members of the second species that were absent in the plastome analysis. Consequently, Wu et al. (2013) suggested that *Hesperocnide* be subsumed in the genus *Urtica*, since these two genera show some morphological similarities. However, owing to this equivocality about the phylogeny of *Hesperocnide*, we suggest a more rigorous examination of this genus to fully validate its status.

## Conclusion and Future Directions

Our study provides important novel insights on Urticeae phylogeny and plastome evolution. The detailed comparative analyses show that Urticeae plastomes exhibit striking differences in genome size, gene number, inversions, intron loss, sequence repeats, and IR/SC boundaries. These kinds of variations will be useful for studies on molecular marker discovery, population genetics, and phylogeny. Resolving the enigmatic relationships within tribe Urticeae has, to date, been a daunting task due to the paucity of genomic resources for the clade. Our study is the first to report phylogenetic relationships in Urticeae based on a broad sampling of whole plastome sequences. This dataset allowed for resolution of several recalcitrant branches (e.g., the relationship of *Poikilospermum* to *Urera*, the sister relationship of *Girardinia*, etc.) that were ambiguous in previous studies. Although our taxon sampling was sufficient to resolve relationships among the major clades in the tribe, additional sampling of particular genera (e.g., *Laportea*) and species (e.g., *Laportea aestuans* and *Hesperocnide sandwicensis*) would further refine our understanding of phylogenetic relationships in Urticeae. Building on the solid framework established here, future studies with even greater taxonomic and genomic sampling could contribute to a better understanding of the diversification patterns in Urticeae in relation to climatic, biogeographic, and ecological factors.

## Data Availability Statement

The datasets presented in this study can be accessed at NCBI GenBank; the list of accessions can be found in Supplementary Table 1.

## Author Contributions

Z-YW, D-ZL, JL, and CO conceptualized the study. Z-YW, JL, RM, AM, and YZ collected the samples. OO and CO conducted the analyses. CO and Z-YW drafted the manuscript. Z-YW, CO, GS, MW, OO, RM, D-ZL, and AM revised the manuscript. All authors read and approved the final manuscript.

## Conflict of Interest

The authors declare that the research was conducted in the absence of any commercial or financial relationships that could be construed as a potential conflict of interest.

## Publisher’s Note

All claims expressed in this article are solely those of the authors and do not necessarily represent those of their affiliated organizations, or those of the publisher, the editors and the reviewers. Any product that may be evaluated in this article, or claim that may be made by its manufacturer, is not guaranteed or endorsed by the publisher.

## Supplementary Material

Supplementary Figure 1 **(A)** Mauve alignment showing gene arrangements within the studied 57 Urticeae plastomes (length indicated above). Large colored boxes represent the gene blocks and the colored lines indicates linear position of different genes in the plastome. **(B)** Comparison of 57 Urticeae CP genomes using mVISTA, with the *E. parvum* genome as the reference. The *y*-axis represents the percent identity within 50–100%. Gray arrows indicate the direction of gene transcription. Blue blocks indicate conserved genes, while red blocks indicate conserved non-coding sequences (CNS).

Supplementary Figure 2 Variable sites in homologous regions of the 57 sampled plastomes from Urticeae. The *y*-axis represent the nucleotide diversity (Pi) of each window, and *x*-axis is the position of the midpoint of each window used in the Sliding window analysis. **(A)** Coding regions. **(B)** Non-coding regions. **(C)** The *ycf*1 gene tree depicting highly resolved and supported relationships achieved by the identified barcode.

Supplementary Figure 3 **(A)** Phylogenetic relationships of Urticeae tribe inferred from maximum likelihood (ML) and Bayesian inference (BI) based on CP coding (CDS) regions. Support values above the branches are maximum likelihood bootstrap support values (ML_BS)/Bayesian posterior probabilities (BI_PP)—note: branches with no support values indicate both ML_BS ≥ 90 and BI_PP = 1.00—whereas “*” indicate incongruence between ML and BI trees. Major clades of Urticeae *s.l.* are indicated on the right, respectively. CDS, chloroplast coding region. **(B)** Phylogenetic relationships of Urticeae tribe inferred from maximum likelihood (ML) and Bayesian inference (BI) based on CP non-coding (nCDS) regions. Support values above the branches are maximum likelihood bootstrap support (ML_BS)/Bayesian posterior probability (BI_PP)—note that branches with no support values indicate both ML_BS ≥ 90 and BI_PP = 1.00—whereas “*” indicate incongruence between ML and BI trees. Major clades of Urticeae *s.l.* are indicated on the right, respectively. nCDS, chloroplast non-coding region. **(C)** Phylogenetic relationships of Urticeae tribe inferred from maximum likelihood (ML) and Bayesian inference (BI) based on integrated CP genome and nrDNA trees. Support values above the branches are maximum likelihood bootstrap support (ML_BS)/Bayesian posterior probability (BI_PP)—note that branches with no support values indicate both ML_BS ≥ 90 and BI_PP = 1.00—whereas “*” indicate incongruence between ML and BI trees. Major clades of Urticeae *s.l.* are indicated on the right, respectively. CP, Complete chloroplast genome; nrDNA, nuclear ribosomal DNA (18S-ITS1-5.8S-ITS2-26S).
