# Supplementary material for: Deep Insights Into the Plastome Evolution and Phylogenetic Relationships of the Tribe Urticeae (Family Urticaceae)
Source: Front Plant Sci. 2022 May 20;13:870949. doi: 10.3389/fpls.2022.870949 (PMC9164014; doi:10.3389/fpls.2022.870949)
Supplement: Supplementary file 1 [file Table_1.docx]

**Supplementary Table S1**

Plant materials examined, their provenance, and accession numbers for respective DNA regions. The species denoted with a ⃰ represent the new sequences. Abbreviations: CP-chloroplast genome, ITS (ITS1-5.8S-ITS2), nrDNA- nuclear ribosomal DNA (18S-ITS1-5.8S-ITS2-26S), and trnL-F - trnL-F intergenic spacer.

| Taxon | Herbarium | Voucher | Country | GenBank accession numbers for each DNA region | | |
| --- | --- | --- | --- | --- | --- | --- |
|  |  |  |  | CP | ITS/nrDNA | *trn*L-F |
| *Dendrocnide*_*basirotunda*_J2078 ⃰ | KUN | 12CS5733 | China | OM761900 | OM892754 | OM761900 |
| *Dendrocnide*_*basirotunda*_ak001 | KUN | 408 | China | ­ | KC284962 | KC285014 |
| *Dendrocnide*_*basirotunda*_ak002 | KUN | 12429 | Laos | ­ | KM586452 | KM586624 |
| *Dendrocnide*_*excels*a_ak003 | NSW 718764 | s.n. |  | ­ | ­ | FJ432265 |
| *Dendrocnide*_*meyeniana*_D2 | KUN | Yi20111184 | Taiwan, China | ­ | KF137838 | KF138311 |
| *Dendrocnide*_*meyeniana*_D7 ⃰ | KUN | WuZY2012428 | Taiwan, China | OM761901 | OM892753 | OM761901 |
| *Dendrocnide*_*meyeniana*_ak004 | KUN | TW-2012247 | Taiwan, China | ­ | KM586432 | KM586604 |
| *Dendrocnide*_*sinuata*_D1 ⃰ | KUN | WuZY-09238 | China | OM761902 | OM892752 | OM761902 |
| *Dendrocnide*_*sinuata*_J7885 ⃰ | KUN | 13CS6832 | China | OM761903 | OM892751 | OM761903 |
| *Dendrocnide*_sp_ak005 | KUN | WuZY-09035 | China | ­ | KF137840 | KF138313 |
| *Dendrocnide*_*stimulans*_ak006 | NSW | 4441 |  | ­ | ­ | FJ432274 |
| *Dendrocnide*_*urentissima*_D4 ⃰ | KUN | GBOWS0461 | China | OM761904 | OM892750 | OM761904 |
| *Dendrocnide*_*urentissima*_J1561 ⃰ | KUN | 12CS3808 | China | OM761905 | OM892749 | OM761905 |
| *Dendrocnide*_*urentissima*_ak007 | KUN | 409 | China | ­ | KC284963 | KC285015 |
| *Discocnide*_*mexicana*_W268 ⃰ | K | 23560 | Mexico | OM877287 | OM892748 | OM877287 |
| *Discocnide*_*mexicana*_ak008 | US | 12763 | Guatemala | ­ | KM586466 | KM586638 |
| *Discocnide*_*mexicana*_ak009 | B | 2205 |  | ­ | ­ | DQ179369 |
| *Giradinia*_*bullosa*_A1 ⃰ | KUN | KE002 | Kenya | OM877286 | OM892745 | OM877286 |
| *Girardinia*_*chingiana*_G1 ⃰ | KUN | liuj-10730 | China | OM877288 | OM892744 | OM877288 |
| *Girardinia*_*chingiana*_G58 ⃰ | KUN | liuj-10775 | China | OM877285 | OM892743 | OM877285 |
| *Girardinia*_*chingiana*_G59 ⃰ | KUN | liuj-10717 | China | OM877274 | OM892742 | OM877274 |
| *Girardinia*_*diversifolia*_G10 ⃰ | KUN | WuZY-09013 | Rwanda | OM877289 | OM892740 | OM877289 |
| *Girardinia*_*diversifolia*_G61 ⃰ | KUN | RC1817 | Nepal | OM877271 | OM892738 | OM877271 |
| *Girardinia*_*diversifolia*_A2 ⃰ | KUN | KE001 | Kenya | OM877273 | OM892741 | OM877273 |
| *Girardinia*_*diversifolia*_G56 ⃰ | KUN | WuZY-09012 | China | OM877272 | OM892739 | OM877272 |
| *Girardinia*_*formosana*_*Hayata*_G3 ⃰ | KUN | GLM-103143 | China | OM877270 | OM892737 | OM877270 |
| *Girardinia*_*formosana*_*Hayata*_G31 ⃰ | KUN | GLM-103093 | China | OM877269 | OM892736 | OM877269 |
| *Girardinia*_*suborbiculata*_subsp_*grammata*_G22 ⃰ | KUN | WuZY-09349 | China | OM877268 | OM892734 | OM877268 |
| *Girardinia*_*suborbiculata*_subsp._*grammata_*G21 ⃰ | KUN | WuZY-09338 | China | MT465765 | OM892735 | MT465765 |
| *Girardinia*_*suborbiculata*_subsp_*suborbiculata*_G51 ⃰ | KUN | WuZY-10012 | China | OM877266 | OM892732 | OM877266 |
| *Girardinia*_*suborbiculata*_subsp_*suborbiculata*_G53 ⃰ | KUN | WuZY-10010 | China | OM877265 | OM892731 | OM877265 |
| *Girardinia*_*suborbiculata*_subsp._*suborbiculata*_G15 ⃰ | KUN | WuZY-10008 | China | OM877267 | OM892733 | OM877267 |
| *Girardinia*_*suborbiculata*_subsp_*triloba*_G19 ⃰ | KUN | WuZY-10089 | China | OM877264 | OM892730 | OM877264 |
| *Girardinia*_*suborbiculata*_subsp_*triloba*_G30 ⃰ | KUN | WuZY-10070 | China | OM877262 | OM892728 | OM877262 |
| *Girardinia*_*suborbiculata*_subsp._*triloba*_G24 ⃰ | KUN | WuZY-10357 | China | OM877263 | OM892729 | OM877263 |
| *Hesperocnide*_*tenella*_W61 ⃰ | E | E00850023 | USA | OM761913 | OM892725 | OM761913 |
| *Hesperocnide*_*tenella*_W277 ⃰ | K | 23571 | California | MN189964 | OM892724 | MN189964 |
| *Hesperocnide*_*tenella*_ak011 | B | 57086 | USA | ­ | KF558907 | KF559027 |
| *Hesperocnide*_*tenella*_ak012 | BSB | 12535-C | USA | ­ | KF558930 | KF559050 |
| *Hesperocnide*_*sandwicensis*_ak013 | EA | 16765 | Kenya | ­ | KM586429 | KM586601 |
| *Hesperocnide*_*sandwicensis*_ak014 | US | 1464 | Argentina | ­ | KM586458 | KM586630 |
| *Hesperocnide*_*sandwicensis*_ak015 | US | 19975 | USA | ­ | KM586459 | KM586631 |
| *Laportea*_*aestuans*_L30 ⃰ | KUN | Bruno12 | Brazil | OM761915 | OM892723 | OM761915 |
| *Laportea*_*aestuans*_W166 ⃰ | FLAS | FLAS233360 | USA | OM761916 | OM892721 | OM761916 |
| *Laportea*_*aestuaus*_W116 ⃰ | K | 7604 | Bolivia | OM761917 | OM892722 | OM761917 |
| *Laportea*_*alatipes*_ak016 | EA | 1204 | Tanzania | ­ | KM586434 | KM586606 |
| *Laportea*_*alatipes*_ak017 | EA | 11331 | Tanzania | ­ | KM586447 | KM586619 |
| *Laportea*_*bulbifera*_GLGE14842 ⃰ | KUN | GLGE14842 | China | OM761918 | OM892720 | OM761918 |
| *Laportea*_*bulbifera*_S12787 ⃰ | KUN | 11CS3716 | China | OM761919 | OM892719 | OM761919 |
| *Laportea*_*bulbifera*_L5 | KUN | WuZY-09423 | China | ­ | KF137870 | KF138349 |
| *Laportea*_*canadensis*_W167 ⃰ | FLAS | FLAS238497 | USA | OM761920 | OM892718 | OM761920 |
| *Laportea*_*canadensis*_ak018 | B | 2959 | USA | ­ | KF971188 | KF971221 |
| *Laportea*_*cuspidata*_L27 ⃰ | K | TianXH263 | China | OM761921 | OM892717 | OM761921 |
| *Laportea*_*cuspidata*_S11822 ⃰ | KUN | 10CS2361 | China | OM761922 | OM892716 | OM761922 |
| *Laportea*_*cuspidata*_ak019 | BSB | s.n. | India | ­ | KX271353 | KX271434 |
| *Laportea*_*decumana*_L15 ⃰ | K | C8746 | Indonesia | OM877281 | OM892816 | OM877281 |
| *Laportea*_*grossa*_L2 ⃰ | KUN | WH01 | South Africa | OM877280 | OM892759 | OM877280 |
| *Laportea*_*interrupta*_ak020 | BSB | s.n. | India | ­ | KX271354 | KX271435 |
| *Laportea*_*interrupta*_ak021 | EA | 2502 | Kenya | ­ | KM586445 | KM586617 |
| *Laportea*_*interrupta*_ak022 | EA | 2218 | Tanzania | ­ | KM586446 | KM586618 |
| *Laportea*_*lanceolata*_ak023 | EA | 9152 | Tanzania | ­ | KM586433 | KM586605 |
| *Laportea*_*medogensis*_GLGE141037 ⃰ | KUN | GLGE141037 | China | OM877279 | OM892760 | OM877279 |
| *Laportea*_*mooreana*_L12 ⃰ | K | 14811 | Unknown | OM761923 | OM892761 | OM761923 |
| *Laportea*_*ovalifolia*_L14 ⃰ | K | 951 | Nigeria | OM761924 | OM892762 | OM761924 |
| *Laportea*_*peduncularis*_ak024 | BSB | 8713 | South Africa | ­ | KF558927 | KF559047 |
| *Laportea*_*ruderalis*_ak025 | US | 6 | Federated States of Micronesia | ­ | KM586461 | KM586633 |
| *Laportea*_*ruderalis*_ak026 | US | 14300 | Guam | ­ | KM586462 | KM586634 |
| *Laportea*_sp_ak027 | NSW | 4497 |  | ­ | ­ | FJ432263 |
| *Nanocnide*_*japonica*_N1 | KUN | Liuj-10735 | China | ­ | KF137879 | KF138359 |
| *Nanocnide*_*japonica*_N3 ⃰ | KUN | liuj-10746 | China | OM761926 | OM892764 | OM761926 |
| *Nanocnide*_*japonica*_N4 | KUN | Liuj-10749 | China | ­ | KF137880 | KF138360 |
| *Nanocnide*_*lobata*_J2527 ⃰ | KUN | GanQL278 | China | OM761927 | OM892765 | OM761927 |
| *Nanocnide*_*lobata*_N6 ⃰ | KUN | liuj-10799 | China | OM761928 | OM892766 | KF138362 |
| *Nanocnide*_*lobata*_ak028 | K | 2091 | Japan | ­ | KF971189 | KF971222 |
| *Obetia*_*aldabrensis*_W291 ⃰ | K | 1358 | Seychelles | OM761929 | OM892767 | OM761929 |
| *Obetia*_*aldabrensis*_ak029 | US | 1357 | Seychelles | ­ | KM586460 | KM586632 |
| *Obetia*_*carruthersiana*_ak030 | B | 1112 | Namibia | ­ | KF971187 | KF971220 |
| *Obetia*_*pinnatifida*_ak031 | EA | 11371 | Tanzania | ­ | KM586449 | KM586621 |
| *Obetia*_*radula*_W292 ⃰ | K | 326 | Tanzania | OM877290 | OM892768 | OM877290 |
| *Obetia*_*radula*_W294 ⃰ | K | 26424 | Tanzania | OM877291 | OM892769 | OM877291 |
| *Obetia*_*radula*_ak032 | B | 341 | Tanzania | ­ | KX271352 | KX271433 |
| *Obetia*_*tenax*_ak033 | K | 9 | South Africa | ­ | KF137886 | KF138367 |
| *Poikilospermum*_*cordifolium*_Poi7 ⃰ | K | CWL.1 | Singapore | OM761930 | OM892772 | OM761930 |
| *Poikilospermum*_*lanceolatum*_Poi8 ⃰ | KUN | 12CS4965 | China | OM761931 | OM892773 | OM761931 |
| *Poikilospermum*_*lanceolatum*_S4494 ⃰ | KUN | S4494 | China | OM761932 | OM892774 | OM761932 |
| *Poikilospermum*_*naucleiflorum*_Poi6 ⃰ | K | 145 | Burma | OM877282 | OM892775 | OM877282 |
| *Poikilospermum*_sp_ak037 | KUN | 13176 | Laos | ­ | KM586453 | KM586625 |
| *Poikilospermum*_sp_ak038 | KUN | 12578 | Laos | ­ | KM586454 | KM586626 |
| *Poikilospermum*_sp_ak039 | KUN | 12766 | Laos | ­ | KM586455 | KM586627 |
| *Poikilospermum*_*suaveolens*_Pi2 ⃰ | KUN | GBOWS736 | China | OM877293 | OM892776 | KF138397 |
| *Poikilospermum*_*suaveolens*_Pi3 | KUN | 9160 | China | ­ | KF137914 | KF138398 |
| *Poikilospermum*_*suaveolen*s_ak040 | KUN | 13870 | China | ­ | KM586456 | KM586628 |
| *Touchardia*_*latifolia*_T1 | KUN | Jffrey201101 | USA | ­ | KF137927 | KF138412 |
| *Touchardia*_*latifolia*_T2 ⃰ | KUN | Jffrey201102 | Hawaii | OM761934 | OM892778 | OM761934 |
| *Urera*_*alceifolia*_ak041 | BM | Monro 4346 |  | ­ | ­ | KF138413 |
| *Urera*_*baccifera*_Ur21 ⃰ | K | 9103 | Bolivia | OM761935 | OM892779 | OM761935 |
| *Urera*_*baccifera*_ak046 | US | 385 | Brazil | ­ | KM586468 | KM586640 |
| *Urera*_*baccifera*_ak047 | US | 3930 | Brazil | ­ | KM586469 | KM586641 |
| *Urera*_*baccifera*_W155 ⃰ | K | 2479 | Planted in Kew | OM877294 | OM892780 | OM877294 |
| *Urera*_*batesii*_ak048 | K | 3412 | Equatorial Guinea | ­ | KF971186 | KF971219 |
| *Urera*_*cameroonensis*_Ur12 ⃰ | K | 2605 | Ondo province | OM877278 | OM892781 | OM877278 |
| *Urera*_*capitata*_W143 ⃰ | K | 7608 | Bolivia | OM877277 | OM892782 | OM877277 |
| *Urera*_*caracasana*_ak049 | US | 3748 | Mexico | ­ | KM586467 | KM586639 |
| *Urera*_*caracasana*_ak050 | K | 8834 | Bolivia | ­ | KF137929 | KF138415 |
| *Urera*_cf_*cordifolia*_Ur15 ⃰ | K | 2486 | West Africa | OM877276 | OM892783 | OM877276 |
| *Urera*_sp_W158 ⃰ | K | 6619 | Cuba | OM877295 | OM892815 | OM877295 |
| *Urera*_*elata*_ak051 | US | 1694 | Mexico | ­ | KM586471 | KM586643 |
| *Urera*_*elata*_ak052 | US | 2224 | Panama | ­ | KM586470 | KM586642 |
| *Urera*_*fischeri*_ak055 | EA | 85/22 | Kenya | ­ | KM586427 | KM586599 |
| *Urera*_*fischeri*_ak056 | EA | 14648 | Kenya | ­ | KM586443 | KM586615 |
| *Urera*_*glabra*_Ur1 | KUN | 100694 | Hawaii | ­ | KF137930 | KF138416 |
| *Urera*_*glabra*_Ur17 ⃰ | K | 8701 | Hawaii | OM761936 | OM892784 | OM761936 |
| *Urera*_*glabra*_ak058 | BISH | 1673 | ­ | ­ | ­ | AF501614 |
| *Urera*_*lianoides*_ak064 | BM | Solano 6825 | Costa Rica | ­ | ­ | KF138418 |
| *Urera*_*hypselodendron*_Ur16 ⃰ | K | IM86 | Sudan | OM761937 | OM892785 | OM761937 |
| *Urera*_*hypselodendron*_ak066 | EA | 95 | Kenya | ­ | KM586450 | KM586622 |
| *Urera*_*hypselodendron*_ak067 | EA | 12404 | Tanzania | ­ | KM586439 | KM586611 |
| *Urera*_*oligoloba*_Ur23 ⃰ | K | 18461 | Madagascar | OM877275 | OM892786 | OM877275 |
| *Urera*_*robusta*_Ur19 ⃰ | K | 5398 | Colony Province, Gold coast | OM761938 | OM892787 | OM761938 |
| *Urera*_*sansibarica*_ak069 | EA | 11527 | Tanzania | ­ | KM586428 | KM586600 |
| *Urera*_*sansibarica*_ak070 | EA | 97 | Kenya | ­ | KM586444 | KM586616 |
| *Urera*_sp_W144 ⃰ | K | 7466 | ­ | OM877296 | OM892814 | OM877296 |
| *Urera*_*trinervis*_ak073 | C | 3920 | Ethiopia | ­ | KF137932 | KF138420 |
| *Urera*_*trinervis*_ak074 | EA | 12513 | Democratic Republic of Congo | ­ | KM586440 | KM586612 |
| *Urtica*_*andicola*_ak075 | BSB | 276 | Peru | ­ | KF558940 | KF559060 |
| *Urtica*_*angustifolia*_J3303 ⃰ | KUN | Liuetal432 | China | OM761939 | OM892788 | OM761939 |
| *Urtica*_*angustifolia*_ak076 | MO | 30 | Mongolia | ­ | KF558902 | KF559022 |
| *Urtica*_*angustifolia*_ak077 | B | 1603 | Mongolia | ­ | KF971217 | KF971250 |
| *Urtica*_*angustifolia*_ak078 | B | 8694 | Khazachstan | ­ | KX271379 | KX271456 |
| *Urtica*_*ardens*_GLGE152058 ⃰ | KUN | GLGE152058 | China | OM761940 | OM892789 | OM761940 |
| *Urtica*_*ardens*_S12891 ⃰ | KUN | GLGE141122 | China | OM761941 | OM892790 | OM761941 |
| *Urtica*_*ardens*_ak079 | B | 8684-4 | China | ­ | KX271400 | KX271476 |
| *Urtica*_*aspera*_ak080 | CHR 511575 | s.n. | New Zealand | ­ | KX271374 | ­ |
| *Urtica*_*atrichocaulis*_S11193 ⃰ | KUN | YNS0188 | China | OM761942 | OM892791 | OM761942 |
| *Urtica*_*atrichocauli*s_ak081 | HUH | 90389 | China | ­ | KX271378 | KX271455 |
| *Urtica*_*atrovirens*_ak082 | BSB | 7798 | Italy | ­ | KF558895 | KF559016 |
| *Urtica*_*atrovirens*_ak083 | B | 7800 | Italy | ­ | KX271390 | KX271466 |
| *Urtica*_*australis*_ak084 | CHR 496662 | 368/93 |  | ­ | KX271371 | ­ |
| *Urtica*_*berteroana*_ak085 | B | s.n. | Chile | ­ | KX271384 | KX271460 |
| *Urtica*_*berteroana*_ak086 | MO | s.n. | Chile | ­ | KX271383 | KX271459 |
| *Urtica*_*bianorii*_ak087 | B | 8155 | Spain | ­ | KF558917 | KF559037 |
| *Urtica*_*cannabina*_ak088 | MO | 322 | China | ­ | KX271370 | KX271451 |
| *Urtica*_*cannabina*_ak089 | BSB | 8678 | Mongolia | ­ | KF558923 | KF559043 |
| *Urtica*_*chamaedryoides*_W162 ⃰ | FLAS | 5034 | USA | OM761943 | OM892792 | OM761943 |
| *Urtica*_*chamaedryoides*_W173 ⃰ | FLAS | FLAS204629 | USA | OM761944 | OM892793 | OM761944 |
| *Urtica*_*chamaedryoides*_ak090 | BM | 299 | Mexico | ­ | KF971202 | KF971235 |
| *Urtica*_*circularis*_ak091 | GB | 135 | Brazil | ­ | KX271386 | KX271462 |
| *Urtica*_*circularis*_ak092 | B | 9311 | Brazil | ­ | KF971200 | KF971233 |
| *Urtica*_*dioica*_subsp_*dioca*_ak093 | B | 2002/2b | SPAIN | ­ | KF558920 | KF559040 |
| *Urtica*_*dioica*_J5488 ⃰ | KUN | YangYP-Q-4177 | China | OM761945 | OM892794 | OM761945 |
| *Urtica*_*dioica*_subsp._*xijiangensis*_U41 ⃰ | KUN | 1024 | China | MT465760 | OM892795 | MT465760 |
| *Urtica*_*dioica*_W174 ⃰ | FLAS | FLAS219395 | USA | OM761947 | OM892796 | OM761947 |
| *Urtica*_*domingensis*_W145 ⃰ | K | 8540 | Dominican Republic | OM761948 | OM892797 | OM761948 |
| *Urtica*_*domingensis*_ak094 | B | 3820 | Dominican Republic | ­ | KX271414 | ­ |
| *Urtica*_*echinata*_ak095 | B | 5852 | Peru | ­ | KF558886 | KF559007 |
| *Urtica*_*echinata*_ak096 | B | 7279 | Peru | ­ | KF558944 | KF559064 |
| *Urtica*_*echinata*_ak097 | GB | 11657 | Ecuador | ­ | KX271427 | KX271501 |
| *Urtica*_f*erox*_ak098 | B | 8211 | New Zealand | ­ | KF558904 | KF559024 |
| *Urtica*_*fissa*_ak099 | B | 8129 | Taiwan, China | ­ | KX271397 | KX271473 |
| *Urtica*_*fissa*_ak100 | MO | 951 | China | ­ | KF558905 | KF559025 |
| *Urtica*_*fissa*_ak101 | HUH | 1135 | China | ­ | ­ | KX271471 |
| *Urtica*_*flabellata*_ak102 | B | 7728 | Peru | ­ | KF971199 | KF971232 |
| *Urtica*_*flabellata*_ak103 | B | 8819 | Peru | ­ | KF558908 | KF559028 |
| *Urtica*_*fragilis*_ak104 | MO | 5216 | Lebanon | ­ | KX271403 | KX271478 |
| *Urtica*_*fragilis*_ak105 | B | 2410-C | Turkey | ­ | KX271404 | KX271479 |
| *Urtica*_*glomerulaeflora*_ak106 | GB | 478 | Chile | ­ | KX271381 | KX271457 |
| *Urtica*_*glomerulaeflora*_ak107 | K | s.n. | Chile | ­ | KX271382 | KX271458 |
| *Urtica*_*gracilenta*_ak108 | BM | 6277 | Mexico | ­ | KF971201 | KF971234 |
| *Urtica*_*gracilenta*_ak109 | TEX | 901 | Mexico | ­ | KF971197 | KF971230 |
| *Urtica*_*gracilis*_Ait_subsp_*aquatica*_ak110 | B | 31 | Guatemala | ­ | KF971214 | KF971247 |
| *Urtica*_*gracilis*_subsp_*aquatica*_ak111 | B | 5847 | Peru | ­ | KF558896 | KF559017 |
| *Urtica*_*gracilis*_Ait_subsp_*gracilis*_ak112 | K | 9916 | USA | ­ | KF971216 | KF971249 |
| *Urtica*_*gracilis*_Ait_subsp_*holosericea*_ak113 | TEX | 2002-330 | Mexico | ­ | KF971215 | KF971248 |
| *Urtica*_*gracilis*_Ait_subsp_*mollis*_ak114 | CONC | 81901 | Chile | ­ | KF558935 | KF559055 |
| *Urtica*_*grandidentata*_ak115 | K | s.n. | Indonesia | ­ | KX271402 | KX271477 |
| *Urtica*_*grandidentata*_ak116 | K | 37901B | Indonesia | ­ | KX271401 | ­ |
| *Urtica*_*himalayensis*_ak117 | K | 590 | India | ­ | KY284027 | ­ |
| *Urtica*_*hyperborea*_J5455 ⃰ | KUN | YangYP-Q-2111 | China | OM761949 | OM892798 | OM761949 |
| *Urtica*_*hyperborea*_ak118 | M | 140 | China | ­ | KX271364 | KX271445 |
| *Urtica*_*hyperborea*_ak119 | MO | 716 | China | ­ | KX271365 | KX271446 |
| *Urtica*_*incisa*_ak120 | BONN | s.n. | Australia | ­ | KX271375 | KX271452 |
| *Urtica*_*incisa*_ak121 | B | 17739 | Australia | ­ | KX271376 | KX271453 |
| *Urtica*_*incisa*_ak122 | NZ Landcare Research | CHR 234516A | New Zealand | ­ | KF971218 | KF971251 |
| *Urtica*_*incisa*_ak123 | BSB | 8212 | New Zealand | ­ | KF558932 | KF559052 |
| *Urtica*_*kioviensis*_U24 ⃰ | K | Unknown | Slovakia | OM761950 | OM892799 | OM761950 |
| *Urtica*_*kioviensis*_ak124 | BSB | 333 | Germany | ­ | KF558924 | KF559044 |
| *Urtica*_*laetevirens*_subsp_*laetevirens*_ak128 | B | 6868 | Russia | ­ | KX271406 | KX271481 |
| *Urtica*_*laetevirens*_subsp_*laetevirens*_ak129 | HUH | s.n. | China | ­ | KX271411 | KX271486 |
| *Urtica*_*laetevirens*_subsp_*laetevirens*_ak130 | LE | 326/1 | Russia | ­ | KX271407 | KX271482 |
| *Urtica*_*lalibertadensis*_ak134 | B | 7896 | Peru | ­ | KF558893 | KF559014 |
| *Urtica*_*leptophylla*_ak135 | B | 7706 | Peru | ­ | KF558892 | KF559013 |
| *Urtica*_*leptophylla*_ak136 | B | 139 | Ecuador | ­ | KX271424 | KX271498 |
| *Urtica*_*leptophylla*_ak137 | MO | 2177 | Venezuela | ­ | KX271428 | KX271502 |
| *Urtica*_*lobulata*_ak138 | PRE | 8709 | RSA | ­ | KX271363 | KX271444 |
| *Urtica*_*longispica*_ak139 | B | 05 | Peru | ­ | KF558899 | KF559020 |
| *Urtica*_*macbridei*_ak140 | B | 9106 | Ecuador | ­ | KX271423 | KX271497 |
| *Urtica*_*macrorrhiza*_U50 ⃰ | KUN | WuZY-10037 | China | MT465759 | OM892800 | MT465759 |
| *Urtica*_*magellanica*_ak141 | SGO | 2912 | Chile | ­ | KF971207 | KF971240 |
| *Urtica*_*magellanica*_ak142 | BM | 2260 | Chile | ­ | KF558933 | KF559053 |
| *Urtica*_*magellanica*_ak143 | CONC | 2873 | Chile | ­ | KX271421 | KX271495 |
| *Urtica*_*magellanica*_U33 ⃰ | K | 4306 | Peru | OM761951 | OM892801 | OM761951 |
| *Urtica*_*mairei*_J1664 ⃰ | KUN | 12CS4575 | China | OM761952 | OM892802 | OM761952 |
| *Urtica*_*mairei*_ak144 | BSB | 5247 | Tibet, China | ­ | KX271398 | KX271474 |
| *Urtica*_*mairei*_var_*mairei*_Q20 ⃰ | KUN | LIUJIE | ­ | OM877284 | OM892803 | OM877284 |
| *Urtica*_*masafuerae*_ak145 | GB | s.n. | Chile | ­ | KX271380 | ­ |
| *Urtica*_*massaica*_ak146 | B | 1050 | Uganda | ­ | KX271388 | KX271464 |
| *Urtica*_*massaica*_ak147 | B | 52 | Tanzania | ­ | KX271389 | KX271465 |
| *Urtica*_*massaica*_ak148 | EA | 1109 | Kenya | ­ | KM586424 | KM586596 |
| *Urtica*_*membranifolia*_S13031 ⃰ | KUN | GLM-102742 | China | OM761953 | OM892804 | OM761953 |
| *Urtica*_*membranacea*_ak149 | B | 8252 | Spain | ­ | KX271362 | KX271443 |
| *Urtica*_*membranacea*_ak150 | BSB | s.n. | Italy | ­ | KF558913 | KF559033 |
| *Urtica*_*membranacea*_ak151 | B | 1 | Portugal | ­ | KF558918 | KF559038 |
| *Urtica*_*mexicana*_ak152 | BM | 4821 | Mexico | ­ | KX271420 | KX271494 |
| *Urtica*_*mexicana*_ak153 | MO | 45624 | Mexico | ­ | KX271419 | KX271493 |
| *Urtica*_*mexicana*_ak154 | MO | 791 | Mexico | ­ | KF971195 | KF971228 |
| *Urtica*_*minutifolia*_ak155 | MO | 19695 | Argentina | ­ | KX271432 | KX271506 |
| *Urtica*_*minutifolia*_ak156 | MO | 22338 | Argentina | ­ | KX271430 | KX271504 |
| *Urtica*_*minutifolia*_ak157 | MO | - | Argentina | ­ | KX271431 | KX271505 |
| *Urtica*_*morifolia*_U200 ⃰ | KUN | M2 | Spain | OM761954 | OM892805 | OM761954 |
| *Urtica*_*morifolia*_ak158 | B | s.n. | Spain | ­ | KX271393 | KX271469 |
| *Urtica*_*morifolia*_ak159 | MADJ | 8711 | Portugal | ­ | KX271394 | KX271470 |
| *Urtica*_*neubaueri*_ak160 | M | 1610 | Afghanistan | ­ | KX271356 | KX271437 |
| *Urtica*_*papuana*_ak161 | K | 9129 | Papua New Guinea | ­ | KX271377 | KX271454 |
| *Urtica*_*perconfusa*_ak162 | CHR 536516 | s.n. | New Zealand | ­ | KX271372 | ­ |
| *Urtica*_*parviflora*_ak163 | BONN | s.n. | India | ­ | KY284024 | KY284032 |
| *Urtica*_*peruviana*_ak164 | B | 7625 | Peru | ­ | KF558897 | KF559018 |
| *Urtica*_*pilulifera*_ak165 | B | 8153-C | Spain | ­ | KF558915 | KF559035 |
| *Urtica*_*pilulifera*_ak166 | B | 8120 | Italy | ­ | KX271357 | KX271438 |
| *Urtica*_*pilulifera*_ak167 | B | s.n. | Malta | ­ | KF558916 | KF559036 |
| *Urtica*_*platyphylla*_ak168 | B | s.n. | Japan | ­ | KX271391 | KX271467 |
| *Urtica*_*platyphylla*_ak169 | B | 357 | Japan | ­ | KF558945 | KF559065 |
| *Urtica*_*portosanctana*_ak170 | B | 8234 | Spain | ­ | KX271360 | KX271441 |
| *Urtica*_*portosanctana*_ak171 | Herb Kurschner | 13279 | Portugal | ­ | KF558929 | KF559049 |
| *Urtica*_*pseudomagellanica*_ak172 | BSB | 728- C | Bolivia | ­ | KX271425 | KX271499 |
| *Urtica*_*radicans*_U21 ⃰ | K | 1987 | Unknown | OM877283 | OM892806 | OM877283 |
| *Urtica*_*rupestris*_U28 ⃰ | KUN | 177 | Avola vechhia | OM761955 | OM892807 | OM761955 |
| *Urtica*_*rupestris*_ak173 | PALERMO | s.n. | Italy | ­ | KX271392 | KX271468 |
| *Urtica*_*simensis*_ak174 | GB | 1995 | Ethiopia | ­ | KX271387 | KX271463 |
| *Urtica*_*spatulata*_ak175 | MO | 1338 | Brazil | ­ | KX271385 | KX271461 |
| *Urtica*_*spirealis*_ak176 | MO | 99.7516 | Guatemala | ­ | KX271417 | KX271491 |
| *Urtica*_*spirealis*_ak177 | TEX | 27 | Mexico | ­ | KF971196 | KF971229 |
| *Urtica*_sp_U18 | KUN | Lixinhui-1102 | Kenya | ­ | KF137941 | KF138429 |
| *Urtica*_sp_U19 ⃰ | KUN | LL-2011 | Australia | OM761956 | OM892808 | KF138430 |
| *Urtica*_*stachyoides*_ak178 | M | 261 | Spain | ­ | KX271358 | KX271439 |
| *Urtica*_*stachyoides*_ak179 | B | 8230 | Spain | ­ | KF558906 | KF559026 |
| *Urtica*_*subincisa*_ak180 | BM | 4901 | Mexico | ­ | KX271415 | KX271489 |
| *Urtica*_*subincisa*_ak181 | MO | 459 | Mexico | ­ | KF971203 | KF971236 |
| *Urtica*_*sykesii*_ak182 | CHR 511586 | 97/3 | New Zealand | ­ | KX271373 | ­ |
| *Urtica*_*taiwaniana*_ak183 | BSB | 00573-C |  | ­ | KF558925 | KF559045 |
| *Urtica*_*taiwaniana*_ak184 | KUN | 11903 | China | ­ | KM586422 | KM586594 |
| *Urtica*_*taiwaniana*_ak185 | KUN | 11345 | China | ­ | KM586420 | KM586592 |
| *Urtica*_*thunbergiana*_J2498 ⃰ | KUN | GanQL168 | China | OM761957 | OM892809 | OM761957 |
| *Urtica*_*thunbergiana*_subsp_*thunbergiana*_ak186 | BONN | s.n. | Japan | ­ | KX271405 | KX271480 |
| *Urtica*_*thunbergiana*_subsp_*thunbergiana*_ak187 | HUH | 250 | Japan | ­ | KY284040 | KY284046 |
| *Urtica*_*triangularis*_subsp_*triangularis* _ak188 | MO | 2576 | China | ­ | KX271367 | KX271448 |
| *Urtica*_*triangularis*_ak189 | KUN | 5801 | China | ­ | KC284958 | KC285010 |
| *Urtica*_*triangularis*_ak190 | KUN | 12157 | China | ­ | KC284959 | KC285011 |
| *Urtica*_*triangularis*_ak191 | KUN | 1339 | China | ­ | KM586404 | KM586576 |
| *Urtica*_*triangularis*_subsp_*pinnatifida*_ak192 | MO | 631 | China | ­ | KX271369 | KX271450 |
| *Urtica*_*triangularis*_subsp_*pinnatifida*_ak193 | KUN | 80860 | China | ­ | KF137943 | KF138431 |
| *Urtica*_*triangularis*_subsp_*pinnatifida*_ak194 | MO | 2354 | China | ­ | KX271368 | KX271449 |
| *Urtica*_*trichantha*_ak195 | B | 7821 | Peru | ­ | KX271429 | KX271503 |
| *Urtica*_*trichantha*_ak196 | BSB | 8848 | Peru | ­ | KX271426 | KX271500 |
| *Urtica*_*trichantha*_ak197 | B | 3326 | Peru | ­ | KF558946 | KF559066 |
| *Urtica*_*urens*_ak198 | B | 8231 | Spain | ­ | KX271359 | KX271440 |
| *Urtica*_*urens*_W175 ⃰ | FLAS | FLAS166382 | USA | OM761959 | OM892810 | OM761959 |
| *Urtica*_*urens*_ak199 | B | 5667 | Germany | ­ | KF558889 | KF559010 |
| *Urtica*_*urens*_U203 ⃰ | KUN | U2 | Spain | OM761958 | ­ | OM761958 |
| *Urtica*_*urentivelutina*_ak200 | B | 7907 | Peru | ­ | KF558898 | KF559019 |
| *Urtica*_*zayuensis*_ak201 | KUN | 10133 | China | ­ | KF137944 | KF138432 |
| *Urtica*_*zayuensis*_ak202 | KUN | 10361 | China | ­ | KF137945 | KF138433 |
| *Zhengyia*_*shennongensis*_Zh1 ⃰ | KUN | Liuj14656 | China | OM761908 | OM892811 | OM761908 |
| *Zhengyia*_*shennongensis*_Zh5 ⃰ | KUN | Liuj14660 | China | OM761909 | OM892812 | OM761909 |
| *Zhengyia*_*shennongensis*_Zh11 ⃰ | KUN | Liuj14666 | China | OM761910 | OM892813 | OM761910 |
| *Cannabis*_*sativa*_ct203 | KUN | 2012-126 | China | ­ | KM586391 | KM586563 |
| *Cannabis*_*sativa*_ct204 |  |  |  | MH118118 | ­ | MH118118 |
| *Humulus*_*lupulus*_ct205 | KUN | 201108110012 | China | ­ | KM586403 | KM586575 |
| *Humulus*_*lupulus*_ct206 | - | - | - | NC_028032 | ­ | NC_028032 |
| *Ficus*_*hirta*_ct207 | - | - | - | MT934444 | ­ | MT934444 |
| *Ficus*_*hirta*_ct208 | - | CYR-1 | China | MN364706 | ­ | MN364706 |
| *Boehmeria*_*tomentosa*_B38 ⃰ | KUN | WuZY-09011 | China | MN189945 | OM892757 | KF138291 |
| *Debregeasia*_*elliptica*_De19 ⃰ | KUN | WuZY-10134 | China | MN189948 | OM892755 | MN189948 |
| *Elatostema*_*parvum*_E7 ⃰ | KUN | WuZY-09214 | China | OM761906 | OM892747 | KF138326 |
| *Pilea*_*alpina*_W118 ⃰ | KUN | 8482 | China | OM877292 | OM892771 | OM877292 |
| *Rousselia*_*humilis*_W141 ⃰ | K | 8274 | Dominican Republic | OM761933 | OM892777 | OM761933 |
| *Forsskaolea*_*angustifolia*_W149 ⃰ | K | 2878 | Planted in Kew | MT465767 | OM892746 | MT465767 |
| *Parietaria*_*micrantha*_Pa1 ⃰ | KUN | WuZY-10373 | China | MN189966 | OM892770 | MN189966 |
| *Australina*_*pusilla*_Au6 ⃰ | K | 5574 | New Zealand | OM761896 | OM892758 | OM761896 |
| *Gyrotaenia*_*microcarpa*_Gy5 ⃰ | K | 68859 | Mexico | OM761911 | OM892727 | OM761911 |
| *Gyrotaenia*_*myriocarpa*_W115 ⃰ | K | 7479 | Dominican Republic | OM761912 | OM892726 | OM761912 |
| *Gyrotaenia*_*myriocarpa*_ak010 | US | 12971 | Dominica | ­ | KM586472 | KM586644 |
| *Cecropia*_*pachystachya*_W55 ⃰ | KUN | Bruno5-2017 | Brazil | OM761898 | OM892756 | - |
| *Maoutia*_*puya*_M5 ⃰ | KUN | MY613 | China | OM761925 | OM892763 | - |
