# Supplementary material for: Deep Insights Into the Plastome Evolution and Phylogenetic Relationships of the Tribe Urticeae (Family Urticaceae)
Source: Front Plant Sci. 2022 May 20;13:870949. doi: 10.3389/fpls.2022.870949 (PMC9164014; doi:10.3389/fpls.2022.870949)
Supplement: Supplementary file 2 [file Table_2.docx]

**Supplementary Table S2**

The characteristics of the six Urticeae datasets. Abbreviations: CP-chloroplast genome, CDS- chloroplast coding region, nCDS- chloroplast non-coding region, nrDNA- nuclear ribosomal DNA (18S-ITS1-5.8S-ITS2-26S), CP+nrDNA- choloroplast genome+ nuclear ribosomal DNA, trnL-F + ITS- trnL-F intergenic spacer + ITS locus data.

| **DNA region** | **Aligned length (bp)** | **No. of variable parsimony-uninformative sites** | **No. of parsimony-informative sites** | **Tree length** | **Consistency index (CI)** | **Retention index (RI)** | **Rescaled consistency index (RC)** | **Model** | **ML optimization likelihood** | **BI estimated marginal likelihood** |
| --- | --- | --- | --- | --- | --- | --- | --- | --- | --- | --- |
| **CP** | 218896 | 16389 | 48586 | 143102 | 0.644 | 0.902 | 0.581 | GTR+I+G | -1140477.939282 | -1148085.27 |
| **CDS** | 88442 | 5891 | 18027 | 50195 | 0.641 | 0.908 | 0.582 | GTR+I+G | -435003.826868 | -439423.80 |
| **nCDS** | 153520 | 15198 | 31667 | 101239 | 0.661 | 0.894 | 0.591 | GTR+G | -734473.485060 | -734598.87 |
| **nrDNA** | 6062 | 358 | 982 | 5033 | 0.434 | 0.809 | 0.351 | GTR+I+G | -34034.741529 | -35852.94 |
| **CP+nrDNA** | 224958 | 16747 | 49568 | 148257 | 0.636 | 0.899 | 0.572 | GTR+I+G | -1178439.447847 | -1187401.11 |
| ***trnL-F* + ITS** | 1949 | 151 | 903 | 5099 | 0.395 | 0.903 | 0.356 | GTR+I+G | -27296.926551 | -27862.18 |
