# Supplementary material for: Deep Insights Into the Plastome Evolution and Phylogenetic Relationships of the Tribe Urticeae (Family Urticaceae)
Source: Front Plant Sci. 2022 May 20;13:870949. doi: 10.3389/fpls.2022.870949 (PMC9164014; doi:10.3389/fpls.2022.870949)
Supplement: Supplementary file 3 [file Table_3.docx]

**Supplementary Table S3**

The distribution of the four repeat types (dispersed, palindromic, tandem and SSR-simple sequence repeat) across Urticeae plastomes. Abbreviations: F-forward, R-reverse, and C- complement repeats.

| **Species** | **Dispersed** | | | | **Palindromic** | **Tandem** | **SSR** | **Total** |
| --- | --- | --- | --- | --- | --- | --- | --- | --- |
|  | **F** | **R** | **C** | **Total** |  |  |  |  |
| *Dendrocnide basirotunda*_J2078 | 16 | 0 | 0 | 16 | 20 | 33 | 64 | 133 |
| *Dendrocnide meyenia*_D7 | 24 | 2 | 0 | 26 | 25 | 29 | 65 | 145 |
| *Dendrocnide sinuata*_J7885 | 19 | 4 | 0 | 23 | 22 | 34 | 56 | 135 |
| *Dendrocnide urentissima*_D4 | 16 | 0 | 0 | 16 | 20 | 34 | 64 | 134 |
| *Discocnide mexicana*_W268 | 27 | 13 | 10 | 50 | 40 | 34 | 70 | 194 |
| *Girardinia bullosa*_A1 | 25 | 1 | 0 | 26 | 29 | 11 | 50 | 116 |
| *Girardinia chingiana*_G1 | 21 | 0 | 0 | 21 | 20 | 20 | 49 | 110 |
| *Girardinia diversifolia*_G56 | 43 | 0 | 0 | 43 | 26 | 29 | 40 | 138 |
| *Girardinia formosana hayata*_G3 | 20 | 0 | 0 | 20 | 24 | 18 | 48 | 110 |
| *Girardinia suborbiculata* subsp *grammata*_G22 | 28 | 0 | 0 | 28 | 24 | 21 | 47 | 120 |
| *Girardinia suborbiculata* subsp *suborbiculata*_G15 | 38 | 0 | 0 | 38 | 23 | 29 | 48 | 138 |
| *Girardinia suborbiculata* subsp *triloba*_G19 | 34 | 0 | 0 | 34 | 25 | 28 | 40 | 127 |
| *Hesperocnide tenella*_W61 | 30 | 0 | 0 | 30 | 15 | 24 | 47 | 116 |
| *Laportea aestuans*_L30 | 15 | 4 | 2 | 21 | 15 | 26 | 50 | 112 |
| *Laportea bulbifera*_GLGE14842 | 9 | 0 | 0 | 9 | 17 | 14 | 30 | 70 |
| *Laportea canadensis*_W167 | 7 | 1 | 0 | 8 | 16 | 7 | 37 | 68 |
| *Laportea cuspidata*_L27 | 8 | 0 | 0 | 8 | 17 | 16 | 18 | 59 |
| *Laportea decumana*_L15 | 16 | 0 | 0 | 16 | 25 | 21 | 49 | 111 |
| *Laportea grossa*_L2 | 16 | 1 | 1 | 18 | 26 | 25 | 82 | 151 |
| *Laportea medogensis*_GLGE141037 | 27 | 26 | 5 | 58 | 31 | 20 | 41 | 150 |
| *Laportea mooreana*_L12 | 36 | 4 | 1 | 41 | 22 | 25 | 46 | 134 |
| *Laportea ovalifolia*_L14 | 15 | 2 | 0 | 17 | 18 | 29 | 48 | 112 |
| *Nanocnide japonica*_N3 | 8 | 0 | 0 | 8 | 10 | 7 | 38 | 63 |
| *Nanocnide lobata*_N6 | 8 | 0 | 0 | 8 | 15 | 8 | 56 | 87 |
| *Obetia aldabrensis*_W291 | 14 | 5 | 3 | 22 | 21 | 27 | 68 | 138 |
| *Poikilospermum cordifolium*_Poi7 | 17 | 6 | 4 | 27 | 20 | 30 | 62 | 139 |
| *Poikilospermum lanceolatum*_Poi8 | 16 | 4 | 3 | 23 | 19 | 26 | 65 | 133 |
| *Poikilospermum naucleiflorum*_Poi6 | 13 | 1 | 3 | 17 | 18 | 24 | 64 | 123 |
| *Touchardia latifolia*_T2 | 13 | 3 | 1 | 17 | 17 | 45 | 62 | 141 |
| *Urera baccifera*_Ur21 | 21 | 2 | 0 | 23 | 17 | 30 | 75 | 145 |
| *Urera cameroonensis*_Ur12 | 12 | 1 | 1 | 14 | 13 | 16 | 74 | 117 |
| *Urera capitata*_W143 | 15 | 1 | 4 | 20 | 17 | 23 | 75 | 135 |
| *Urera* cf *cordifolia*_Ur15 | 12 | 1 | 1 | 14 | 13 | 15 | 71 | 113 |
| *Urera glabra*_Ur17 | 9 | 5 | 1 | 15 | 15 | 37 | 56 | 123 |
| *Urera hypselodendron*_Ur16 | 12 | 1 | 0 | 13 | 14 | 13 | 73 | 113 |
| *Urera oligoloba*_Ur23 | 8 | 1 | 0 | 9 | 14 | 17 | 69 | 109 |
| *Urera robusta*_Ur19 | 10 | 0 | 1 | 11 | 12 | 13 | 69 | 105 |
| *Urtica angustifolia*_J3303 | 10 | 1 | 0 | 11 | 11 | 18 | 41 | 81 |
| *Urtica ardens*_GLGE152058 | 17 | 0 | 1 | 18 | 19 | 16 | 42 | 95 |
| *Urtica atrichocaulis*_S11193 | 11 | 0 | 0 | 11 | 11 | 14 | 45 | 81 |
| *Urtica chamaedryoides*_W162 | 16 | 0 | 0 | 16 | 12 | 17 | 48 | 93 |
| *Urtica dioica* subsp. *xijiangensis*_U41 | 13 | 0 | 0 | 13 | 11 | 15 | 39 | 78 |
| *Urtica dioica*_W174 | 15 | 1 | 0 | 16 | 11 | 23 | 40 | 90 |
| *Urtica domingensis*_W145 | 10 | 0 | 0 | 10 | 14 | 13 | 44 | 81 |
| *Urtica hyperborea*_J5455 | 12 | 0 | 0 | 12 | 13 | 13 | 41 | 79 |
| *Urtica kioviensis*_U24 | 10 | 1 | 0 | 11 | 11 | 18 | 43 | 83 |
| *Urtica macrorrhiza*_U50 | 11 | 1 | 0 | 12 | 11 | 18 | 41 | 82 |
| *Urtica magellanica*_U33 | 17 | 0 | 0 | 17 | 14 | 15 | 49 | 95 |
| *Urtica mairei*_J1664 | 17 | 0 | 1 | 18 | 19 | 15 | 41 | 93 |
| *Urtica membranifolia*_S13031 | 16 | 0 | 1 | 17 | 18 | 16 | 42 | 93 |
| *Urtica morifolia*_U200 | 15 | 0 | 0 | 15 | 16 | 16 | 41 | 88 |
| *Urtica radicans*_U21 | 9 | 1 | 0 | 10 | 11 | 17 | 38 | 76 |
| *Urtica rupestris*_U28 | 11 | 0 | 1 | 12 | 11 | 15 | 41 | 79 |
| *Urtica* sp_U19 | 16 | 0 | 0 | 16 | 13 | 12 | 50 | 91 |
| *Urtica thunbergiana*_J2498 | 16 | 0 | 1 | 17 | 20 | 19 | 37 | 93 |
| *Urtica urens*_W175 | 16 | 0 | 0 | 16 | 13 | 13 | 45 | 87 |
| *Zhengyia shennongensis*_Zh1 | 30 | 19 | 15 | 64 | 36 | 24 | 45 | 169 |
| **Total species** | **966** | **113** | **61** | **1140** | **1030** | **1185** | **2919** | **6274** |
