# Supplementary material for: Deep Insights Into the Plastome Evolution and Phylogenetic Relationships of the Tribe Urticeae (Family Urticaceae)
Source: Front Plant Sci. 2022 May 20;13:870949. doi: 10.3389/fpls.2022.870949 (PMC9164014; doi:10.3389/fpls.2022.870949)
Supplement: Supplementary file 4 [file Table_4.DOCX]

**Supplementary Table S4**

The six simple sequence repeats (SSRs) in each of the Urticeae plastid genomes.

| **Species** | **mono-nucleotide** | **di-nucleotide** | **tri-nucleotide** | **tetra-nucleotide** | **penta-nucleotide** | **hexa-nucleotide** | **Total** |
| --- | --- | --- | --- | --- | --- | --- | --- |
| *Dendrocnide*_*basirotunda*_J2078 | 57 | 5 | 1 | 1 | 0 | 0 | 64 |
| *Dendrocnide*_*meyeniana*_D7 | 57 | 6 | 2 | 0 | 0 | 0 | 65 |
| *Dendrocnide*_*sinuata*_J7885 | 49 | 5 | 2 | 0 | 0 | 0 | 56 |
| *Dendrocnide*_*urentissima*_D4 | 57 | 5 | 1 | 1 | 0 | 0 | 64 |
| *Discocnide*_*mexicana*_W268 | 63 | 6 | 1 | 0 | 0 | 0 | 70 |
| *Giradinia*_*bullosa*_A1 | 45 | 4 | 1 | 0 | 0 | 0 | 50 |
| *Girardinia*_*chingiana*_G1 | 40 | 7 | 2 | 0 | 0 | 0 | 49 |
| *Girardinia*_*diversifolia*_G56 | 34 | 4 | 2 | 0 | 0 | 0 | 40 |
| *Girardinia*_*formosana*_*Hayata*_G3 | 39 | 7 | 2 | 0 | 0 | 0 | 48 |
| *Girardinia*_*suborbiculata*_subsp_*grammata*_G22 | 40 | 5 | 2 | 0 | 0 | 0 | 47 |
| *Girardinia*_*suborbiculata*_subsp_*suborbiculata*_G15 | 40 | 6 | 2 | 0 | 0 | 0 | 48 |
| *Girardinia*_*suborbiculata*_subsp_*triloba*_G19 | 34 | 4 | 2 | 0 | 0 | 0 | 40 |
| *Hesperocnide*_*tenella*_W61 | 43 | 4 | 0 | 0 | 0 | 0 | 47 |
| *Laportea*_*aestuans*_L30 | 46 | 4 | 0 | 0 | 0 | 0 | 50 |
| *Laportea*_*bulbifera*_GLGE14842 | 27 | 2 | 0 | 0 | 1 | 0 | 30 |
| *Laportea*_*canadensis*_W167 | 36 | 1 | 0 | 0 | 0 | 0 | 37 |
| *Laportea*_*cuspidata*_L27 | 17 | 1 | 0 | 0 | 0 | 0 | 18 |
| *Laportea*_*decumana*_L15 | 41 | 8 | 0 | 0 | 0 | 0 | 49 |
| *Laportea*_*grossa*_L2 | 71 | 9 | 2 | 0 | 0 | 0 | 82 |
| *Laportea*_*medogensis*_GLGE141037 | 37 | 0 | 0 | 2 | 0 | 2 | 41 |
| *Laportea*_*mooreana*_L12 | 42 | 4 | 0 | 0 | 0 | 0 | 46 |
| *Laportea*_*ovalifolia*_L14 | 43 | 5 | 0 | 0 | 0 | 0 | 48 |
| *Nanocnide*_*japonica*_N3 | 37 | 1 | 0 | 0 | 0 | 0 | 38 |
| *Nanocnide*_*lobata*_N6 | 54 | 2 | 0 | 0 | 0 | 0 | 56 |
| *Obetia*_*aldabrensis*_W291 | 58 | 9 | 1 | 0 | 0 | 0 | 68 |
| *Poikilospermum*_*cordifolium*_Poi7 | 55 | 6 | 1 | 0 | 0 | 0 | 62 |
| *Poikilospermum*_*lanceolatum*_Poi8 | 58 | 6 | 1 | 0 | 0 | 0 | 65 |
| *Poikilospermum*_*naucleiflorum*_Poi6 | 56 | 6 | 2 | 0 | 0 | 0 | 64 |
| *Touchardia*_*latifolia*_T2 | 59 | 1 | 2 | 0 | 0 | 0 | 62 |
| *Urera*_*baccifera*_Ur21 | 70 | 5 | 0 | 0 | 0 | 0 | 75 |
| *Urera*_*cameroonensis*_Ur12 | 66 | 7 | 1 | 0 | 0 | 0 | 74 |
| *Urera*_*capitata*_W143 | 63 | 11 | 1 | 0 | 0 | 0 | 75 |
| *Urera*_cf_*cordifolia*_Ur15 | 62 | 8 | 1 | 0 | 0 | 0 | 71 |
| *Urera*_*glabra*_Ur17 | 54 | 1 | 1 | 0 | 0 | 0 | 56 |
| *Urera*_*hypselodendron*_Ur16 | 65 | 7 | 1 | 0 | 0 | 0 | 73 |
| *Urera*_*oligoloba*_Ur23 | 60 | 8 | 1 | 0 | 0 | 0 | 69 |
| *Urera*_*robusta*_Ur19 | 61 | 7 | 1 | 0 | 0 | 0 | 69 |
| *Urtica*_*angustifolia*_J3303 | 38 | 3 | 0 | 0 | 0 | 0 | 41 |
| *Urtica*_a*rdens*_GLGE152058 | 41 | 1 | 0 | 0 | 0 | 0 | 42 |
| *Urtica*_*atrichocaulis*_S11193 | 43 | 2 | 0 | 0 | 0 | 0 | 45 |
| *Urtica*_*chamaedryoides*_W162 | 43 | 5 | 0 | 0 | 0 | 0 | 48 |
| *Urtica*_*dioica*_subsp._*xijiangensis*_U41 | 36 | 3 | 0 | 0 | 0 | 0 | 39 |
| *Urtica*_*dioica*_W174 | 37 | 3 | 0 | 0 | 0 | 0 | 40 |
| *Urtica*_*domingensis*_W145 | 42 | 2 | 0 | 0 | 0 | 0 | 44 |
| *Urtica*_*hyperborea*_J5455 | 38 | 3 | 0 | 0 | 0 | 0 | 41 |
| *Urtica*_*kioviensis*_U24 | 41 | 2 | 0 | 0 | 0 | 0 | 43 |
| *Urtica*_*macrorrhiza*_U50 | 39 | 2 | 0 | 0 | 0 | 0 | 41 |
| *Urtica*_*magellanica*_U33 | 44 | 4 | 1 | 0 | 0 | 0 | 49 |
| *Urtica*_mairei_J1664 | 40 | 1 | 0 | 0 | 0 | 0 | 41 |
| *Urtica*_*membranifolia*_S13031 | 41 | 1 | 0 | 0 | 0 | 0 | 42 |
| *Urtica*_*morifolia*_U200 | 36 | 5 | 0 | 0 | 0 | 0 | 41 |
| *Urtica*_*radicans*_U21 | 35 | 3 | 0 | 0 | 0 | 0 | 38 |
| *Urtica*_*rupestris*_U28 | 38 | 3 | 0 | 0 | 0 | 0 | 41 |
| *Urtica*_sp_U19 | 48 | 2 | 0 | 0 | 0 | 0 | 50 |
| *Urtica*_*thunbergiana*_J2498 | 36 | 1 | 0 | 0 | 0 | 0 | 37 |
| *Urtica*_*urens*_W175 | 43 | 2 | 0 | 0 | 0 | 0 | 45 |
| *Zhengyia*_*shennongensis*_Zh1 | 32 | 13 | 0 | 0 | 0 | 0 | 45 |
| **Total** | **2627** | **248** | **37** | **4** | **1** | **2** | **2919** |
