# Supplementary material for: Deep Insights Into the Plastome Evolution and Phylogenetic Relationships of the Tribe Urticeae (Family Urticaceae)
Source: Front Plant Sci. 2022 May 20;13:870949. doi: 10.3389/fpls.2022.870949 (PMC9164014; doi:10.3389/fpls.2022.870949)
Supplement: Supplementary file 5 [file Table_5.DOCX]

**Supplementary Table S5**

The motifs in each of the six SSRs (simple sequence repeats) categories detected in Urticeae plastid genomes.

| **Species** | **mono-nucleotide** | | |  | **di-nucleotide** | | | | **tri-nucleotide** | | | | |
| --- | --- | --- | --- | --- | --- | --- | --- | --- | --- | --- | --- | --- | --- |
|  | **A** | **T** | **C** | **G** | **AT** | **AG** | **TA** | **TC** | **TAA** | **TAT** | **TTA** | **ATA** | **AAT** |
| *Dendrocnide basirotunda*_J2078 | 16 | 40 | 1 | 0 | 5 | 0 | 0 | 0 | 0 | 0 | 1 | 0 | 0 |
| *D. meyeniana*_D7 | 19 | 36 | 2 | 0 | 6 | 0 | 0 | 0 | 0 | 0 | 2 | 0 | 0 |
| *D. sinuata*_J7885 | 12 | 36 | 1 | 0 | 5 | 0 | 0 | 0 | 0 | 1 | 1 | 0 | 0 |
| *D. urentissima*_D4 | 16 | 40 | 1 | 0 | 5 | 0 | 0 | 0 | 0 | 0 | 1 | 0 | 0 |
| *Discocnide mexicana*_W268 | 24 | 39 | 0 | 0 | 5 | 0 | 1 | 0 | 0 | 0 | 1 | 0 | 0 |
| *Giradinia bullosa*_A1 | 14 | 28 | 2 | 1 | 4 | 0 | 0 | 0 | 0 | 0 | 1 | 0 | 0 |
| *G. chingiana*_G1 | 15 | 22 | 2 | 1 | 6 | 0 | 1 | 0 | 0 | 0 | 1 | 0 | 1 |
| *G. diversifolia*_G56 | 15 | 17 | 1 | 1 | 4 | 0 | 0 | 0 | 0 | 0 | 1 | 0 | 1 |
| *G. formosana Hayata*_G3 | 12 | 22 | 4 | 1 | 6 | 0 | 1 | 0 | 0 | 0 | 1 | 0 | 1 |
| *G. suborbiculata* subsp *grammata*_G22 | 15 | 22 | 2 | 1 | 5 | 0 | 0 | 0 | 0 | 0 | 1 | 0 | 1 |
| *G. suborbiculata* subsp *suborbiculata*_G15 | 14 | 22 | 3 | 1 | 6 | 0 | 0 | 0 | 0 | 0 | 1 | 0 | 1 |
| *G. suborbiculata* subsp *triloba*_G19 | 16 | 16 | 1 | 1 | 4 | 0 | 0 | 0 | 0 | 0 | 1 | 0 | 1 |
| *Hesperocnide tenella*_W61 | 15 | 23 | 2 | 3 | 2 | 0 | 2 | 0 | 0 | 0 | 0 | 0 | 0 |
| *Laportea aestuans*_L30 | 21 | 25 | 0 | 0 | 3 | 0 | 1 | 0 | 0 | 0 | 0 | 0 | 0 |
| *L. bulbifera*_GLGE14842 | 8 | 19 | 0 | 0 | 2 | 0 | 0 | 0 | 0 | 0 | 0 | 0 | 0 |
| *L. canadensis*_W167 | 10 | 26 | 0 | 0 | 1 | 0 | 0 | 0 | 0 | 0 | 0 | 0 | 0 |
| *L. cuspidata*_L27 | 4 | 13 | 0 | 0 | 1 | 0 | 0 | 0 | 0 | 0 | 0 | 0 | 0 |
| *L. decumana*_L15 | 10 | 30 | 1 | 0 | 8 | 0 | 0 | 0 | 0 | 0 | 0 | 0 | 0 |
| *L. grossa*_L2 | 25 | 43 | 2 | 1 | 8 | 0 | 1 | 0 | 1 | 0 | 1 | 0 | 0 |
| *L. medogensis*_GLGE141037 | 9 | 28 | 0 | 0 | 0 | 0 | 0 | 0 | 0 | 0 | 0 | 0 | 0 |
| *L. mooreana*_L12 | 12 | 29 | 1 | 0 | 3 | 0 | 1 | 0 | 0 | 0 | 0 | 0 | 0 |
| *L. ovalifolia*_L14 | 18 | 25 | 0 | 0 | 2 | 0 | 3 | 0 | 0 | 0 | 0 | 0 | 0 |
| *Nanocnide japonica*_N3 | 10 | 25 | 1 | 1 | 1 | 0 | 0 | 0 | 0 | 0 | 0 | 0 | 0 |
| *N. lobata*_N6 | 17 | 35 | 1 | 1 | 2 | 0 | 0 | 0 | 0 | 0 | 0 | 0 | 0 |
| *Obetia aldabrensis*_W291 | 21 | 33 | 3 | 1 | 4 | 0 | 5 | 0 | 0 | 0 | 1 | 0 | 0 |
| *Poikilospermum cordifolium*_Poi7 | 17 | 35 | 3 | 0 | 5 | 0 | 1 | 0 | 0 | 0 | 1 | 0 | 0 |
| *P. lanceolatum*_Poi8 | 20 | 35 | 3 | 0 | 5 | 0 | 1 | 0 | 0 | 0 | 1 | 0 | 0 |
| *P. naucleiflorum*_Poi6 | 19 | 34 | 3 | 0 | 5 | 0 | 1 | 0 | 0 | 0 | 2 | 0 | 0 |
| *Touchardia latifolia*_T2 | 25 | 34 | 0 | 0 | 1 | 0 | 0 | 0 | 0 | 0 | 1 | 1 | 0 |
| *Urera baccifera*_Ur21 | 26 | 40 | 4 | 0 | 4 | 0 | 1 | 0 | 0 | 0 | 0 | 0 | 0 |
| *U. cameroonensis*_Ur12 | 26 | 37 | 2 | 1 | 3 | 1 | 3 | 0 | 0 | 0 | 1 | 0 | 0 |
| *U. capitata*_W143 | 25 | 37 | 1 | 0 | 11 | 0 | 0 | 0 | 0 | 0 | 1 | 0 | 0 |
| *U.* cf *cordifolia*_Ur15 | 24 | 36 | 1 | 1 | 4 | 1 | 3 | 0 | 0 | 0 | 1 | 0 | 0 |
| *U. glabra*_Ur17 | 21 | 33 | 0 | 0 | 1 | 0 | 0 | 0 | 0 | 0 | 1 | 0 | 0 |
| *U. hypselodendron*_Ur16 | 26 | 36 | 2 | 1 | 4 | 0 | 3 | 0 | 0 | 0 | 1 | 0 | 0 |
| *U. oligoloba*_Ur23 | 23 | 34 | 2 | 1 | 6 | 0 | 2 | 0 | 0 | 0 | 1 | 0 | 0 |
| *U. robusta*_Ur19 | 20 | 38 | 2 | 1 | 3 | 0 | 4 | 0 | 0 | 0 | 1 | 0 | 0 |
| *Urtica angustifolia*_J3303 | 11 | 22 | 3 | 2 | 1 | 0 | 2 | 0 | 0 | 0 | 0 | 0 | 0 |
| *U. ardens*_GLGE152058 | 14 | 24 | 1 | 2 | 1 | 0 | 0 | 0 | 0 | 0 | 0 | 0 | 0 |
| *U. atrichocaulis*_S11193 | 14 | 24 | 3 | 2 | 0 | 0 | 2 | 0 | 0 | 0 | 0 | 0 | 0 |
| *U. chamaedryoides*_W162 | 13 | 27 | 2 | 1 | 2 | 0 | 3 | 0 | 0 | 0 | 0 | 0 | 0 |
| *U. dioica* subsp. *xijiangensis*_U41 | 13 | 22 | 1 | 0 | 1 | 0 | 2 | 0 | 0 | 0 | 0 | 0 | 0 |
| *U. dioica*_W174 | 15 | 17 | 3 | 2 | 1 | 0 | 2 | 0 | 0 | 0 | 0 | 0 | 0 |
| *U. domingensis*_W145 | 16 | 23 | 2 | 1 | 1 | 0 | 1 | 0 | 0 | 0 | 0 | 0 | 0 |
| *U. hyperborea*_J5455 | 13 | 20 | 3 | 2 | 1 | 0 | 2 | 0 | 0 | 0 | 0 | 0 | 0 |
| *U. kioviensis*_U24 | 17 | 19 | 3 | 2 | 0 | 0 | 2 | 0 | 0 | 0 | 0 | 0 | 0 |
| *U. macrorrhiza*_U50 | 12 | 22 | 3 | 2 | 0 | 0 | 2 | 0 | 0 | 0 | 0 | 0 | 0 |
| *U. magellanica*_U33 | 15 | 26 | 2 | 1 | 1 | 0 | 3 | 0 | 0 | 0 | 1 | 0 | 0 |
| *U. mairei*_J1664 | 15 | 22 | 1 | 2 | 1 | 0 | 0 | 0 | 0 | 0 | 0 | 0 | 0 |
| *U. membranifolia*_S13031 | 18 | 23 | 0 | 0 | 1 | 0 | 0 | 0 | 0 | 0 | 0 | 0 | 0 |
| *U. morifolia*_U200 | 12 | 24 | 0 | 0 | 2 | 0 | 3 | 0 | 0 | 0 | 0 | 0 | 0 |
| *U. radicans*_U21 | 13 | 18 | 2 | 2 | 1 | 0 | 2 | 0 | 0 | 0 | 0 | 0 | 0 |
| *U. rupestris*_U28 | 11 | 20 | 5 | 2 | 1 | 0 | 2 | 0 | 0 | 0 | 0 | 0 | 0 |
| *U.* sp_U19 | 17 | 26 | 3 | 2 | 1 | 0 | 1 | 0 | 0 | 0 | 0 | 0 | 0 |
| *U. urens*_W175 | 14 | 24 | 3 | 2 | 1 | 0 | 1 | 0 | 0 | 0 | 0 | 0 | 0 |
| *Zhengyia shennongensis*_Zh1 | 15 | 17 | 0 | 0 | 10 | 0 | 3 | 0 | 0 | 0 | 0 | 0 | 0 |
| **Total** | **922** | **1565** | **94** | **46** | **178** | **2** | **68** | **0** | **1** | **1** | **28** | **1** | **6** |

**Supplementary Table S5 cont’d**

Subtypes of each of the six SSRs categories detected in Urticeae plastid genomes

| **Species** | **tetra-nucleotide** | | | | | | **penta-nucleotide** | | | | **hexa-nucleotide** | | | | | |
| --- | --- | --- | --- | --- | --- | --- | --- | --- | --- | --- | --- | --- | --- | --- | --- | --- |
|  | **ATAA** | **ATAG** | **AATA** | **AAAT** | **TTTA** | **CTAT** | **ATAAA** | **TAAAC** | **TAATA** | **ATAAT** | **ATAGAT** | **GTAGAT** | **ATTAAT** | **AATATC** | **CCTAAT** | **TTAGGA** |
| *Dendrocnide basirotunda*_J2078 | 0 | 0 | 0 | 0 | 1 | 0 | 0 | 0 | 0 | 0 | 0 | 0 | 0 | 0 | 0 | 0 |
| *D*. *meyeniana*_D7 | 0 | 0 | 0 | 0 | 0 | 0 | 0 | 0 | 0 | 0 | 0 | 0 | 0 | 0 | 0 | 0 |
| *D*. *sinuata*_J7885 | 0 | 0 | 0 | 0 | 0 | 0 | 0 | 0 | 0 | 0 | 0 | 0 | 0 | 0 | 0 | 0 |
| *D*. *urentissima*_D4 | 0 | 0 | 0 | 0 | 1 | 0 | 0 | 0 | 0 | 0 | 0 | 0 | 0 | 0 | 0 | 0 |
| *Discocnide mexicana*_W268 | 0 | 0 | 0 | 0 | 0 | 0 | 0 | 0 | 0 | 0 | 0 | 0 | 0 | 0 | 0 | 0 |
| *Giradinia bullosa*_A1 | 0 | 0 | 0 | 0 | 0 | 0 | 0 | 0 | 0 | 0 | 0 | 0 | 0 | 0 | 0 | 0 |
| *G*. *chingiana*_G1 | 0 | 0 | 0 | 0 | 0 | 0 | 0 | 0 | 0 | 0 | 0 | 0 | 0 | 0 | 0 | 0 |
| *G*. *diversifolia*_G56 | 0 | 0 | 0 | 0 | 0 | 0 | 0 | 0 | 0 | 0 | 0 | 0 | 0 | 0 | 0 | 0 |
| *G*. *formosana* *Hayata*_G3 | 0 | 0 | 0 | 0 | 0 | 0 | 0 | 0 | 0 | 0 | 0 | 0 | 0 | 0 | 0 | 0 |
| *G*. *suborbiculata* subsp *grammata*_G22 | 0 | 0 | 0 | 0 | 0 | 0 | 0 | 0 | 0 | 0 | 0 | 0 | 0 | 0 | 0 | 0 |
| *G*. suborbiculata subsp *suborbiculata*_G15 | 0 | 0 | 0 | 0 | 0 | 0 | 0 | 0 | 0 | 0 | 0 | 0 | 0 | 0 | 0 | 0 |
| *G*. suborbiculata subsp *triloba*_G19 | 0 | 0 | 0 | 0 | 0 | 0 | 0 | 0 | 0 | 0 | 0 | 0 | 0 | 0 | 0 | 0 |
| *Hesperocnide tenella*_W61 | 0 | 0 | 0 | 0 | 0 | 0 | 0 | 0 | 0 | 0 | 0 | 0 | 0 | 0 | 0 | 0 |
| *Laportea* *aestuans*_L30 | 0 | 0 | 0 | 0 | 0 | 0 | 0 | 0 | 0 | 0 | 0 | 0 | 0 | 0 | 0 | 0 |
| *L*. *bulbifera*_GLGE14842 | 0 | 0 | 0 | 0 | 0 | 0 | 0 | 0 | 0 | 1 | 0 | 0 | 0 | 0 | 0 | 0 |
| *L*. *canadensis*_W167 | 0 | 0 | 0 | 0 | 0 | 0 | 0 | 0 | 0 | 0 | 0 | 0 | 0 | 0 | 0 | 0 |
| *L*. *cuspidata*_L27 | 0 | 0 | 0 | 0 | 0 | 0 | 0 | 0 | 0 | 0 | 0 | 0 | 0 | 0 | 0 | 0 |
| *L*. *decumana*_L15 | 0 | 0 | 0 | 0 | 0 | 0 | 0 | 0 | 0 | 0 | 0 | 0 | 0 | 0 | 0 | 0 |
| *L*. *grossa*_L2 | 0 | 0 | 0 | 0 | 0 | 0 | 0 | 0 | 0 | 0 | 0 | 0 | 0 | 0 | 0 | 0 |
| *L*. *medogensi*s_GLGE141037 | 0 | 0 | 0 | 0 | 1 | 1 | 0 | 0 | 0 | 0 | 0 | 0 | 0 | 0 | 1 | 1 |
| *L*. *mooreana*_L12 | 0 | 0 | 0 | 0 | 0 | 0 | 0 | 0 | 0 | 0 | 0 | 0 | 0 | 0 | 0 | 0 |
| *L*. *ovalifolia*_L14 | 0 | 0 | 0 | 0 | 0 | 0 | 0 | 0 | 0 | 0 | 0 | 0 | 0 | 0 | 0 | 0 |
| *Nanocnide japonica*_N3 | 0 | 0 | 0 | 0 | 0 | 0 | 0 | 0 | 0 | 0 | 0 | 0 | 0 | 0 | 0 | 0 |
| *N*. *lobata*_N6 | 0 | 0 | 0 | 0 | 0 | 0 | 0 | 0 | 0 | 0 | 0 | 0 | 0 | 0 | 0 | 0 |
| *Obetia* *aldabrensis*_W291 | 0 | 0 | 0 | 0 | 0 | 0 | 0 | 0 | 0 | 0 | 0 | 0 | 0 | 0 | 0 | 0 |
| *Poikilospermum cordifolium*_Poi7 | 0 | 0 | 0 | 0 | 0 | 0 | 0 | 0 | 0 | 0 | 0 | 0 | 0 | 0 | 0 | 0 |
| *P*. *lanceolatum*_Poi8 | 0 | 0 | 0 | 0 | 0 | 0 | 0 | 0 | 0 | 0 | 0 | 0 | 0 | 0 | 0 | 0 |
| *P*. *naucleiflorum*_Poi6 | 0 | 0 | 0 | 0 | 0 | 0 | 0 | 0 | 0 | 0 | 0 | 0 | 0 | 0 | 0 | 0 |
| *Touchardia* l*atifolia*_T2 | 0 | 0 | 0 | 0 | 0 | 0 | 0 | 0 | 0 | 0 | 0 | 0 | 0 | 0 | 0 | 0 |
| *Urera baccifera*_Ur21 | 0 | 0 | 0 | 0 | 0 | 0 | 0 | 0 | 0 | 0 | 0 | 0 | 0 | 0 | 0 | 0 |
| *U*. *cameroonensis*_Ur12 | 0 | 0 | 0 | 0 | 0 | 0 | 0 | 0 | 0 | 0 | 0 | 0 | 0 | 0 | 0 | 0 |
| *U*. *capitata*_W143 | 0 | 0 | 0 | 0 | 0 | 0 | 0 | 0 | 0 | 0 | 0 | 0 | 0 | 0 | 0 | 0 |
| *U*. cf *cordifolia*_Ur15 | 0 | 0 | 0 | 0 | 0 | 0 | 0 | 0 | 0 | 0 | 0 | 0 | 0 | 0 | 0 | 0 |
| *U*. *glabra*_Ur17 | 0 | 0 | 0 | 0 | 0 | 0 | 0 | 0 | 0 | 0 | 0 | 0 | 0 | 0 | 0 | 0 |
| *U*. *hypselodendron*_Ur16 | 0 | 0 | 0 | 0 | 0 | 0 | 0 | 0 | 0 | 0 | 0 | 0 | 0 | 0 | 0 | 0 |
| *U*. *oligoloba*_Ur23 | 0 | 0 | 0 | 0 | 0 | 0 | 0 | 0 | 0 | 0 | 0 | 0 | 0 | 0 | 0 | 0 |
| *U*. *robusta*_Ur19 | 0 | 0 | 0 | 0 | 0 | 0 | 0 | 0 | 0 | 0 | 0 | 0 | 0 | 0 | 0 | 0 |
| *Urtica* *angustifolia*_J3303 | 0 | 0 | 0 | 0 | 0 | 0 | 0 | 0 | 0 | 0 | 0 | 0 | 0 | 0 | 0 | 0 |
| *U*. *ardens*_GLGE152058 | 0 | 0 | 0 | 0 | 0 | 0 | 0 | 0 | 0 | 0 | 0 | 0 | 0 | 0 | 0 | 0 |
| *U*. a*trichocaulis*_S11193 | 0 | 0 | 0 | 0 | 0 | 0 | 0 | 0 | 0 | 0 | 0 | 0 | 0 | 0 | 0 | 0 |
| *U*. *chamaedryoides*_W162 | 0 | 0 | 0 | 0 | 0 | 0 | 0 | 0 | 0 | 0 | 0 | 0 | 0 | 0 | 0 | 0 |
| *U*. *dioica* subsp. *xijiangensis*_U41 | 0 | 0 | 0 | 0 | 0 | 0 | 0 | 0 | 0 | 0 | 0 | 0 | 0 | 0 | 0 | 0 |
| *U*. *dioica*_W174 | 0 | 0 | 0 | 0 | 0 | 0 | 0 | 0 | 0 | 0 | 0 | 0 | 0 | 0 | 0 | 0 |
| *U*. *domingensis*_W145 | 0 | 0 | 0 | 0 | 0 | 0 | 0 | 0 | 0 | 0 | 0 | 0 | 0 | 0 | 0 | 0 |
| *U*. *hyperborea*_J5455 | 0 | 0 | 0 | 0 | 0 | 0 | 0 | 0 | 0 | 0 | 0 | 0 | 0 | 0 | 0 | 0 |
| *U*. *kioviensis*_U24 | 0 | 0 | 0 | 0 | 0 | 0 | 0 | 0 | 0 | 0 | 0 | 0 | 0 | 0 | 0 | 0 |
| *U*. *macrorrhiza*_U50 | 0 | 0 | 0 | 0 | 0 | 0 | 0 | 0 | 0 | 0 | 0 | 0 | 0 | 0 | 0 | 0 |
| *U*. *magellanica*_U33 | 0 | 0 | 0 | 0 | 0 | 0 | 0 | 0 | 0 | 0 | 0 | 0 | 0 | 0 | 0 | 0 |
| *U*. *mairei*_J1664 | 0 | 0 | 0 | 0 | 0 | 0 | 0 | 0 | 0 | 0 | 0 | 0 | 0 | 0 | 0 | 0 |
| *U*. *membranifolia*_S13031 | 0 | 0 | 0 | 0 | 0 | 0 | 0 | 0 | 0 | 0 | 0 | 0 | 0 | 0 | 0 | 0 |
| *U*. *morifolia*_U200 | 0 | 0 | 0 | 0 | 0 | 0 | 0 | 0 | 0 | 0 | 0 | 0 | 0 | 0 | 0 | 0 |
| *U*. *radicans*_U21 | 0 | 0 | 0 | 0 | 0 | 0 | 0 | 0 | 0 | 0 | 0 | 0 | 0 | 0 | 0 | 0 |
| *U*. *rupestris*_U28 | 0 | 0 | 0 | 0 | 0 | 0 | 0 | 0 | 0 | 0 | 0 | 0 | 0 | 0 | 0 | 0 |
| *U*. sp_U19 | 0 | 0 | 0 | 0 | 0 | 0 | 0 | 0 | 0 | 0 | 0 | 0 | 0 | 0 | 0 | 0 |
| *U*. *thunbergiana*_J2498 | 0 | 0 | 0 | 0 | 0 | 0 | 0 | 0 | 0 | 0 | 0 | 0 | 0 | 0 | 0 | 0 |
| *U*. *urens*_W175 | 0 | 0 | 0 | 0 | 0 | 0 | 0 | 0 | 0 | 0 | 0 | 0 | 0 | 0 | 0 | 0 |
| *Zhengyia shennongensis*_Zh1 | 0 | 0 | 0 | 0 | 0 | 0 | 0 | 0 | 0 | 0 | 0 | 0 | 0 | 0 | 0 | 0 |
| **Total** | **0** | **0** | **0** | **0** | **3** | **1** | **0** | **0** | **0** | **1** | **0** | **0** | **0** | **0** | **1** | **1** |
