# Supplementary figures and images for: Deep Insights Into the Plastome Evolution and Phylogenetic Relationships of the Tribe Urticeae (Family Urticaceae)
Source: Front Plant Sci. 2022 May 20;13:870949. doi: 10.3389/fpls.2022.870949 (PMC9164014; doi:10.3389/fpls.2022.870949)

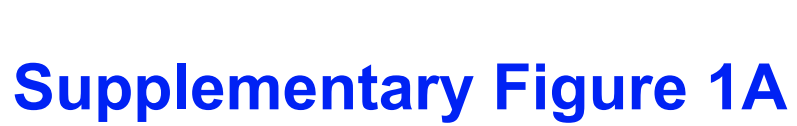

Supplement: Supplementary Figure 1 — (A) Mauve alignment showing gene arrangements within the studied 57 Urticeae plastomes (length indicated above). Large colored boxes represent the gene blocks and the colored lines indicates linear position of different genes in the plastome. (B) Comparison of 57 Urticeae CP genomes using mVISTA, with the E. parvum genome as the reference. The y-axis represents the percent identity within 50–100%. Gray arrows indicate the direction of gene transcription. Blue blocks indicate conserved genes, while red blocks indicate conserved non-coding sequences (CNS). [file Data_Sheet_1.pdf]

Supplementary Figure 2

A

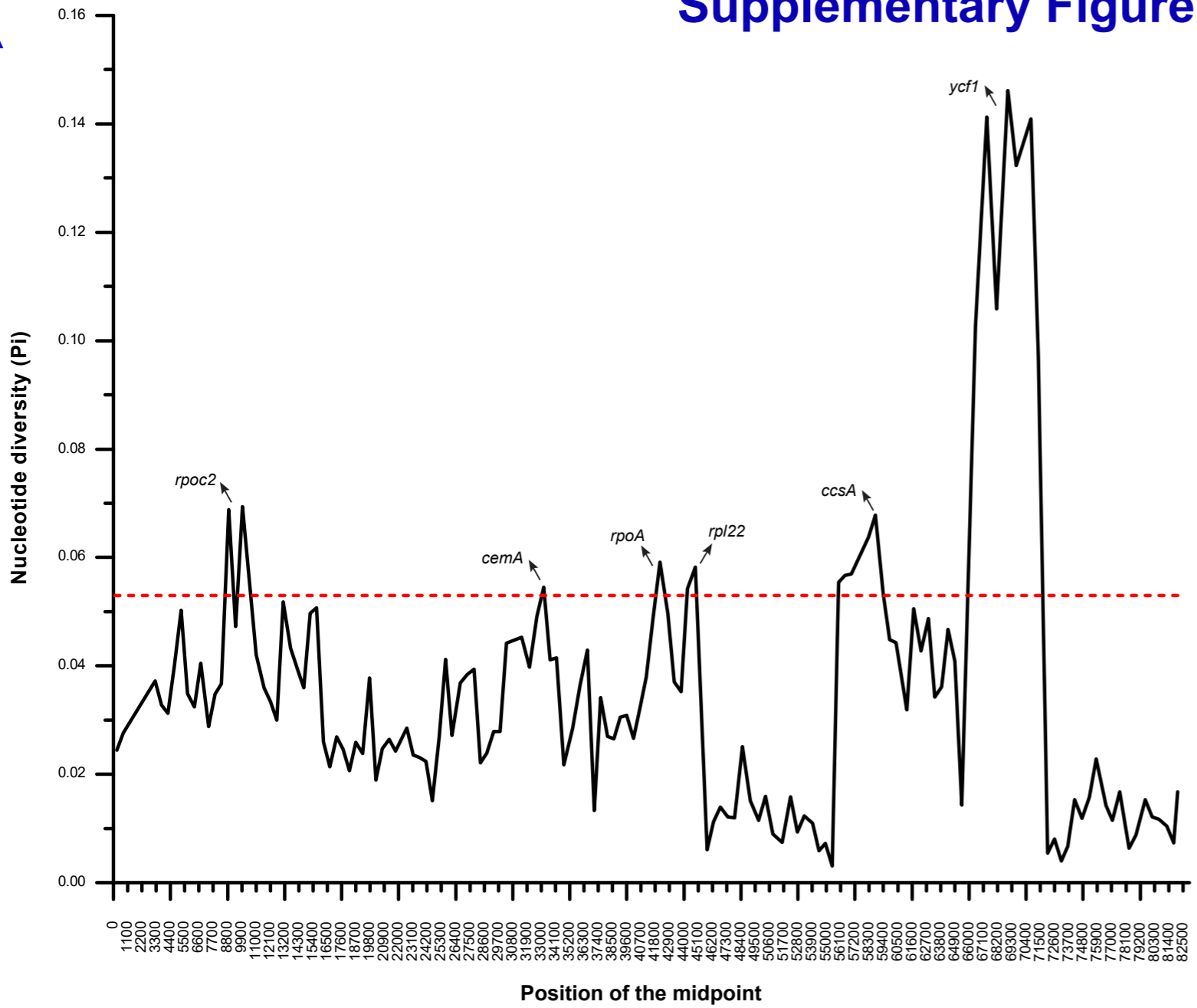

B

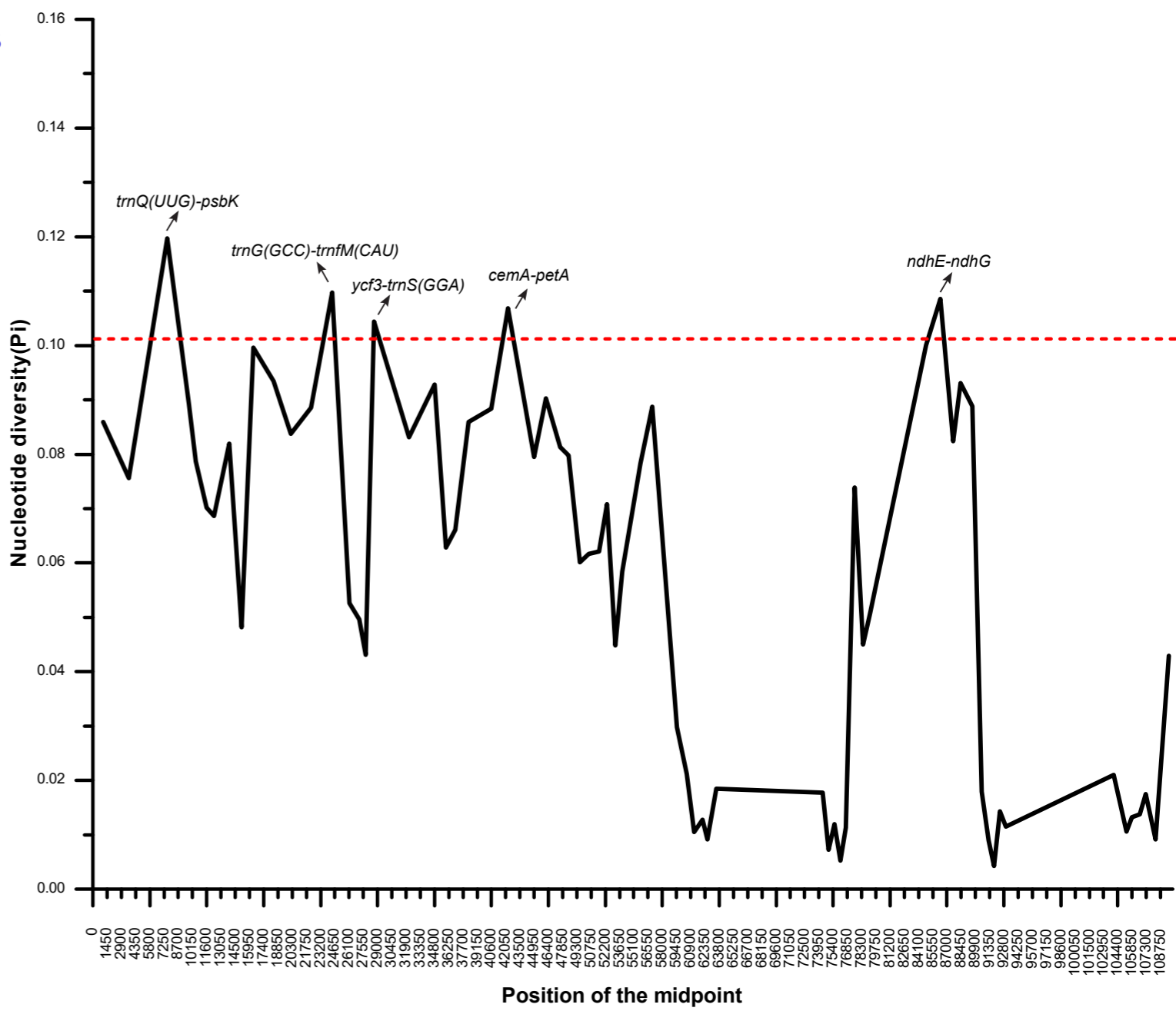

## ycf1 gene tree

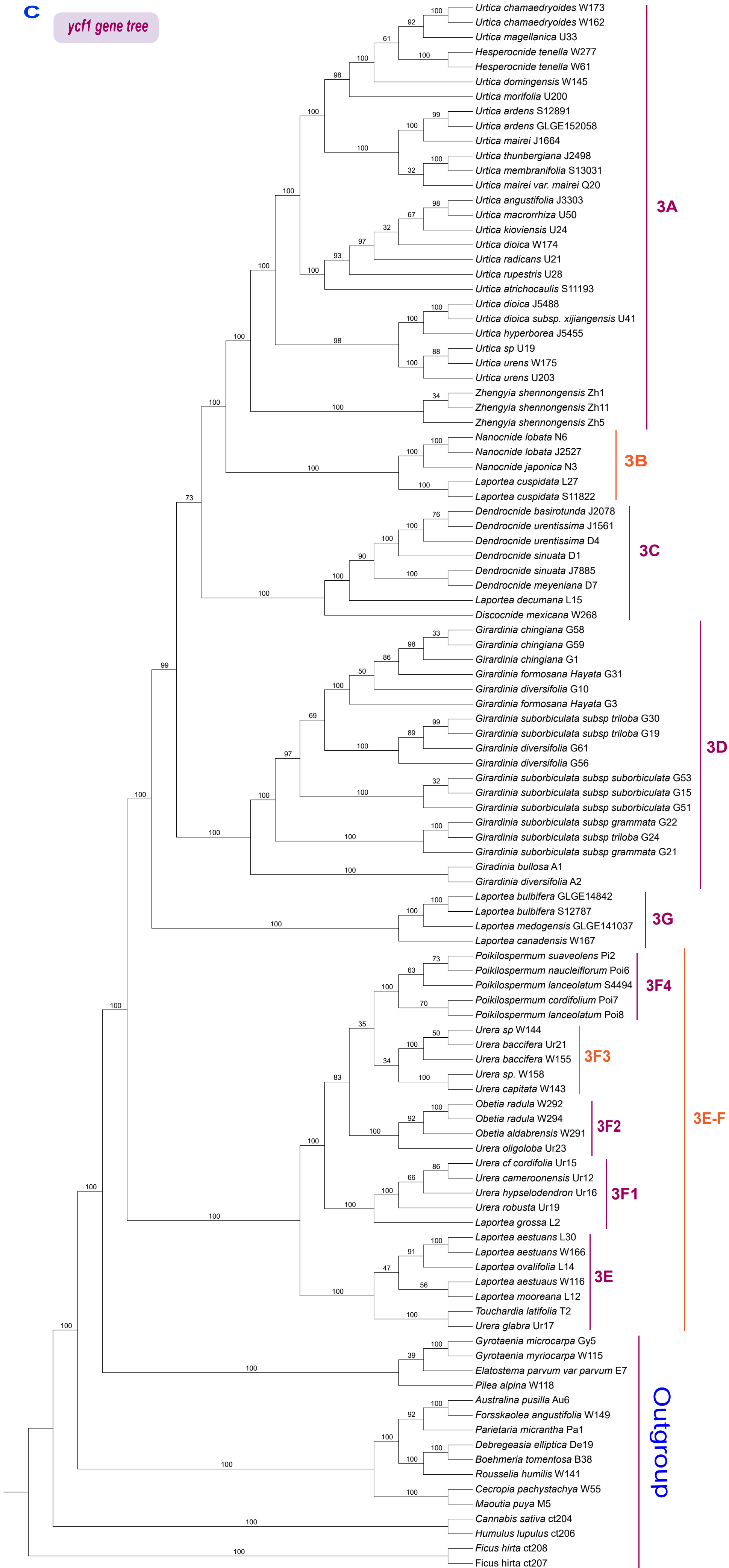

Supplement: Supplementary Figure 2 — Variable sites in homologous regions of the 57 sampled plastomes from Urticeae. The y-axis represent the nucleotide diversity (Pi) of each window, and x-axis is the position of the midpoint of each window used in the Sliding window analysis. (A) Coding regions. (B) Non-coding regions. (C) The ycf1 gene tree depicting highly resolved and supported relationships achieved by the identified barcode. [file Data_Sheet_2.pdf]
