# Supplementary material for: Deep Insights Into the Plastome Evolution and Phylogenetic Relationships of the Tribe Urticeae (Family Urticaceae)
Source: Front Plant Sci. 2022 May 20;13:870949. doi: 10.3389/fpls.2022.870949 (PMC9164014; doi:10.3389/fpls.2022.870949)

Supplementary Figure 3A: CDS

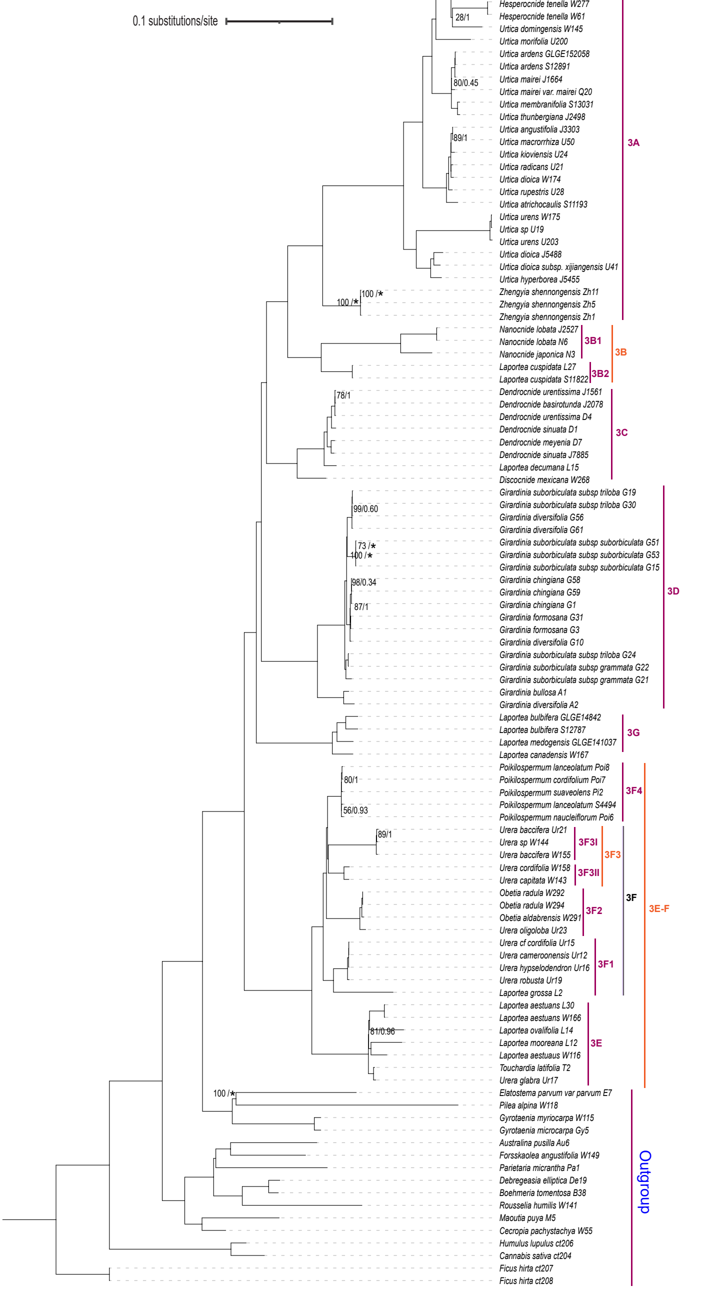

Supplementary Figure 3B: NON-CODING

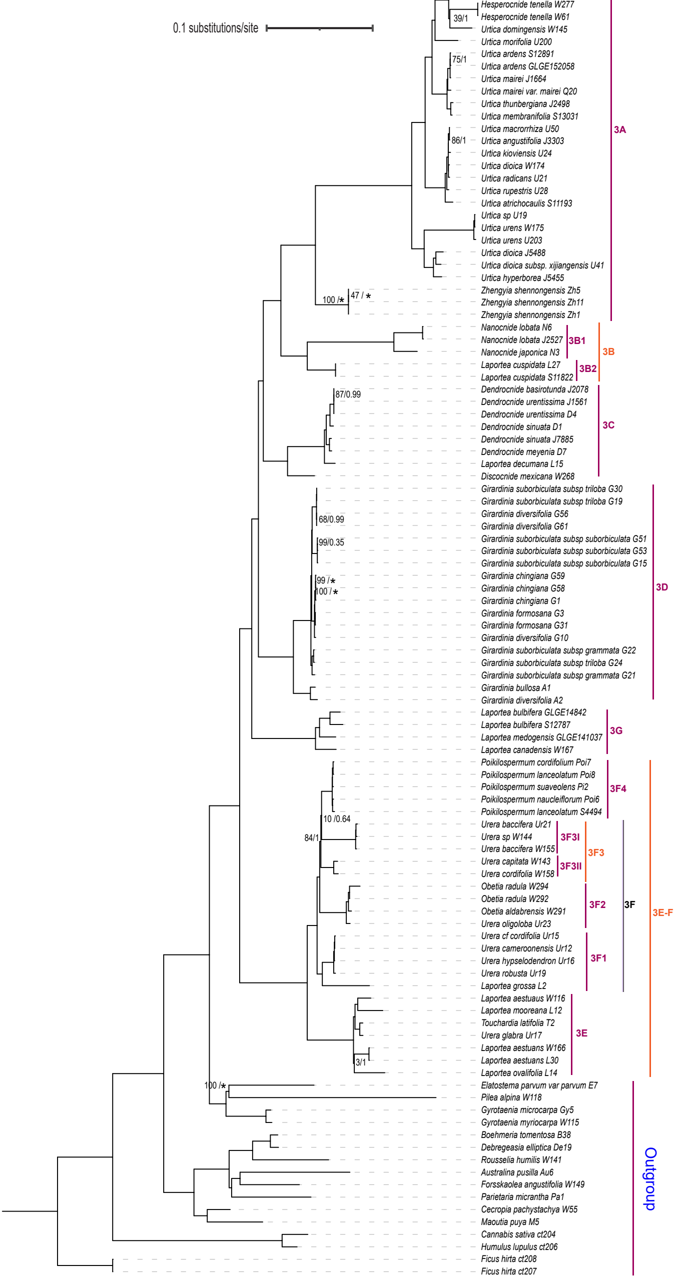

Supplemenary Figure 3C (I): CP

Supplementary Figure 3C (II): nrDNA

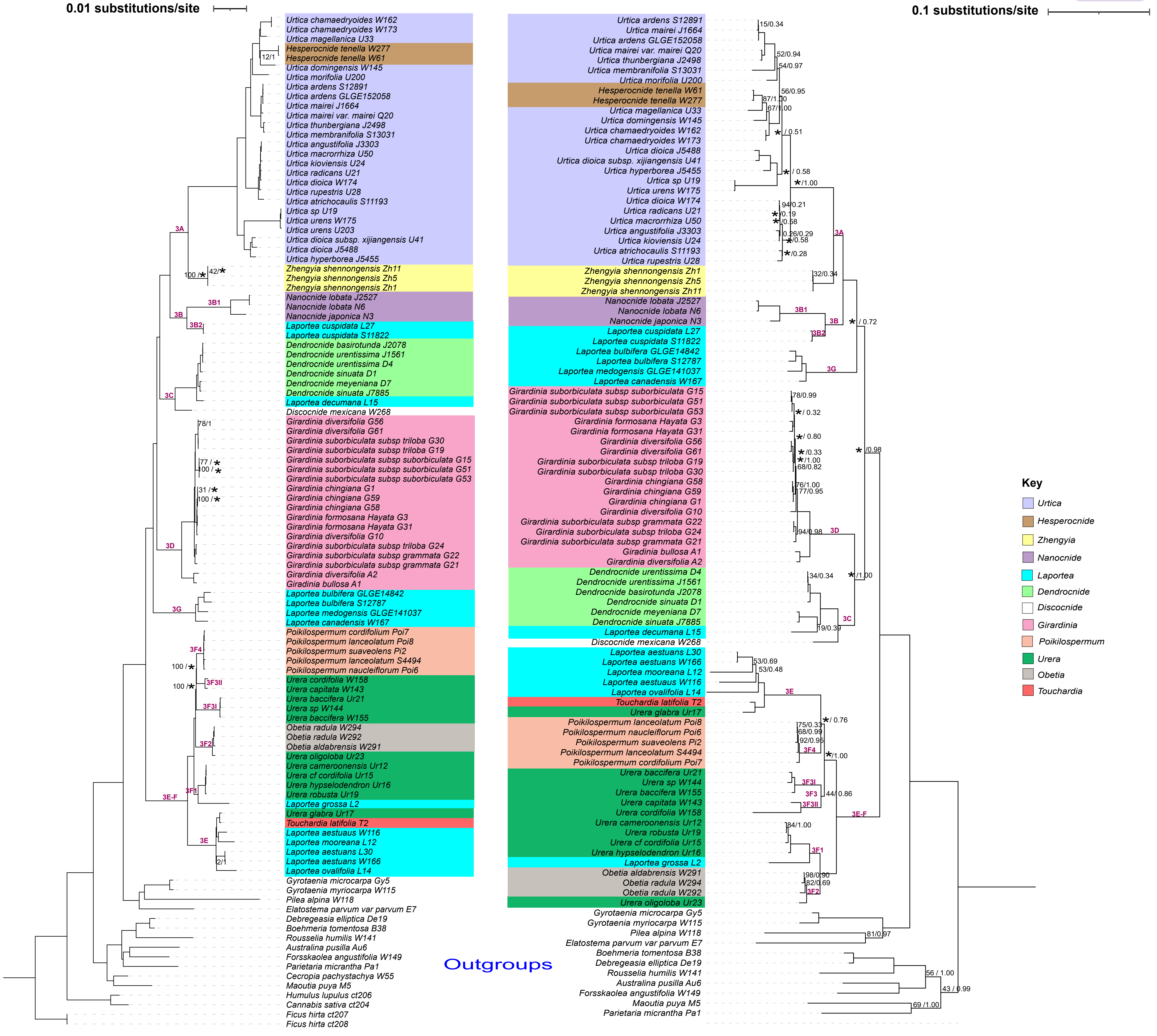

Supplement: Supplementary Figure 3 — (A) Phylogenetic relationships of Urticeae tribe inferred from maximum likelihood (ML) and Bayesian inference (BI) based on CP coding (CDS) regions. Support values above the branches are maximum likelihood bootstrap support values (ML_BS)/Bayesian posterior probabilities (BI_PP)—note: branches with no support values indicate both ML_BS ≥ 90 and BI_PP = 1.00—whereas “*” indicate incongruence between ML and BI trees. Major clades of Urticeae s.l. are indicated on the right, respectively. CDS, chloroplast coding region. (B) Phylogenetic relationships of Urticeae tribe inferred from maximum likelihood (ML) and Bayesian inference (BI) based on CP non-coding (nCDS) regions. Support values above the branches are maximum likelihood bootstrap support (ML_BS)/Bayesian posterior probability (BI_PP)—note that branches with no support values indicate both ML_BS ≥ 90 and BI_PP = 1.00—whereas “*” indicate incongruence between ML and BI trees. Major clades of Urticeae s.l. are indicated on the right, respectively. nCDS, chloroplast non-coding region. (C) Phylogenetic relationships of Urticeae tribe inferred from maximum likelihood (ML) and Bayesian inference (BI) based on integrated CP genome and nrDNA trees. Support values above the branches are maximum likelihood bootstrap support (ML_BS)/Bayesian posterior probability (BI_PP)—note that branches with no support values indicate both ML_BS ≥ 90 and BI_PP = 1.00—whereas “*” indicate incongruence between ML and BI trees. Major clades of Urticeae s.l. are indicated on the right, respectively. CP, Complete chloroplast genome; nrDNA, nuclear ribosomal DNA (18S-ITS1-5.8S-ITS2-26S). [file Data_Sheet_3.pdf]
